# Homogeneous, heterogeneous, and enzyme catalysis in microfluidics droplets

**DOI:** 10.1002/smo.20220001

**Published:** 2023-04-17

**Authors:** Fang Mei, Hongyu Lin, Lianrui Hu, Wei‐Tao Dou, Hai‐Bo Yang, Lin Xu

**Affiliations:** ^1^ Shanghai Key Laboratory of Green Chemistry and Chemical Processes Shanghai Frontiers Science Center of Molecule Intelligent Syntheses School of Chemistry and Molecular Engineering East China Normal University Shanghai China

**Keywords:** enzyme catalysis, heterogeneous catalysis, homogeneous catalysis, microfluidic droplet, supramolecular chemistry

## Abstract

Microfluidics has received extensive attention due to its ability to rapidly prepare a large number of microdroplets with controlled sizes and defined morphologies. In addition to having large surface areas and controllable confinement environments, these prepared microdroplets can be used as analytical detection devices to screen and optimize various kinetic parameters. This review summarizes recent advances in the microfluidic control of droplet‐based catalytic reactions and discusses the role of these droplets in both homogeneous and heterogeneous catalyzes and in the catalysis of macromolecular biological enzymes in water‐in‐oil and oil‐in‐oil environments. Additionally, the existing problems and future development directions of droplets in catalysis are highlighted to promote the development of catalytic reactions in droplet media and provide guidance for the high‐throughput screening of catalysts and the directed evolution of biological enzymes.

## Graphical abstract

Micro/nanofluidics provides a powerful method for controlling the size and shape of synthesized droplets. Thus, it opens a pathway for facilitating microscale chemistry. Here, we summarize recent developments in catalytic reactions occurring in droplets generated by micro/nanofluidics, and highlight the application of droplets in catalytic processes.

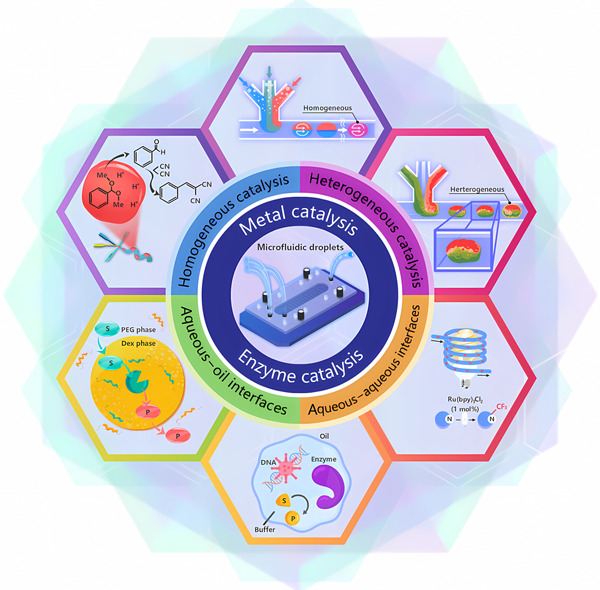

## 1 INTRODUCTION

Microdroplets are widely used as microreactors in a variety of applications, such as biochemical reactions, rapid mixing, and microparticle synthesis.[^1^, ^2^, ^3^] Microfluidic platforms have shifted the paradigm of biochemical experimentation over the past 3 decades.[^4^, ^5^] A key component of this technology is the droplet‐based microfluidic system, which utilizes passive microfluidic structures to quickly produce and control subnanometer‐sized droplets in microchannel environments.[^6^, ^7^] Droplets are formed continuously and robustly through the extrusion and shearing of two mutually immiscible phases in a microchannel. The volumes of these droplets are precisely controlled through variations of the flow rate ratios and channel dimensions. Droplet microfluidics, which is a recently developed technology, involves the generation, manipulation, and application of droplet. Microfluidic droplets typically range from several micrometers to hundreds of micrometers in diameter.^[8]^ During the basic synthetic process of droplets, two incompatible liquids, namely, a continuous phase and a discrete phase, are separately driven into their respective microchannels at a fixed volume flow rate. When the two streams meet at the intersection point, the discrete phase thins gradually and eventually breaks up into droplets due to the shear and squeezing forces exerted by the continuous phase.

Since many biological reactions take place in cells, biological responses in confined environments are essential for life. By internalizing reaction conditions, not only can enzymes react under mild conditions, but also heat and mass transfer are also accelerated, thus increasing the reaction efficiency and productivity from both catalytic and stoichiometric perspectives.^[2]^ To understand biological mechanisms and advance synthetic biology, it is necessary to understand the details of compartmentalized biological reactions. In addition, enzymes are versatile and exquisitely proficient catalysts. Optimized by evolution over time, they can initiate, accelerate, and control a variety of reactions within living systems while ensuring high substrate specificity as well as extraordinary selectivity. Despite their extensive structural and chemical diversity, relatively few natural enzymes have been successfully applied to the treatment of nonliving systems in industrial environments.^[9]^ Because their sizes and relatively isolated environments are similar to those of cells, microfluidic droplets offer a practical platform for performing enzyme‐catalyzed reactions in vitro while retaining the high catalytic activity.^[10]^

As in biological reactions, suitable reagent mixing in microdroplets and the precise control of other finely regulated parameters, including concentration, temperature, and time, are employed for specific organic reactions to increase reaction rates, lessen side effects, and ensure reproducibility. As a result, switching from batch reactors to microdroplets can improve catalytic processes. A catalytic cycle can be carried out in microfluidic droplets with intrinsic safety benefits because only a small number of extremely dangerous, explosive, or hazardous intermediates can be produced in these environments. Additionally, reactants and catalysts can be used in smaller quantities, effectively enabling the screening of a variety of reaction conditions with just small amounts of reagents and catalysts. Numerous studies have demonstrated the benefits of adopting microdroplet technology over batch operations in wet chemical catalysis.[^11^, ^12^, ^13^]

Due to its continuous working mode, superior mixing efficiency, and well‐controlled residence time, microdroplet‐based catalytic technology is facile, versatile, and reproducible.^[14]^ While conventional batch reactors typically require hours or days to complete a catalytic reaction, microdroplets typically require only seconds to tens of minutes.[^15^, ^16^, ^17^] Finally, scaling up is easier in microdroplets than in batch reactors.^[18]^ This can be achieved by scaling up a reactor's operating time or operating several reactors simultaneously, which results in the miniaturization of reactions, incorporating the idea of process intensification and lessening the cost of process scaling up. The synthesis parameters previously optimized in a single‐channel microreactor are maintained during these scale‐up processes, ensuring that the transport/reaction characteristics across numerous microchannels or with a longer reaction time are, in theory, identical. This is advantageous for the quick, dependable, and modular production of synthetic products on a large scale.^[19]^ Throughput can be increased through automation and nonstop 24‐h processing.^[20]^ We can reasonably conclude from the information above that microdroplet technology offers a series of benefits for various catalytic processes.

**FIGURE 1:**
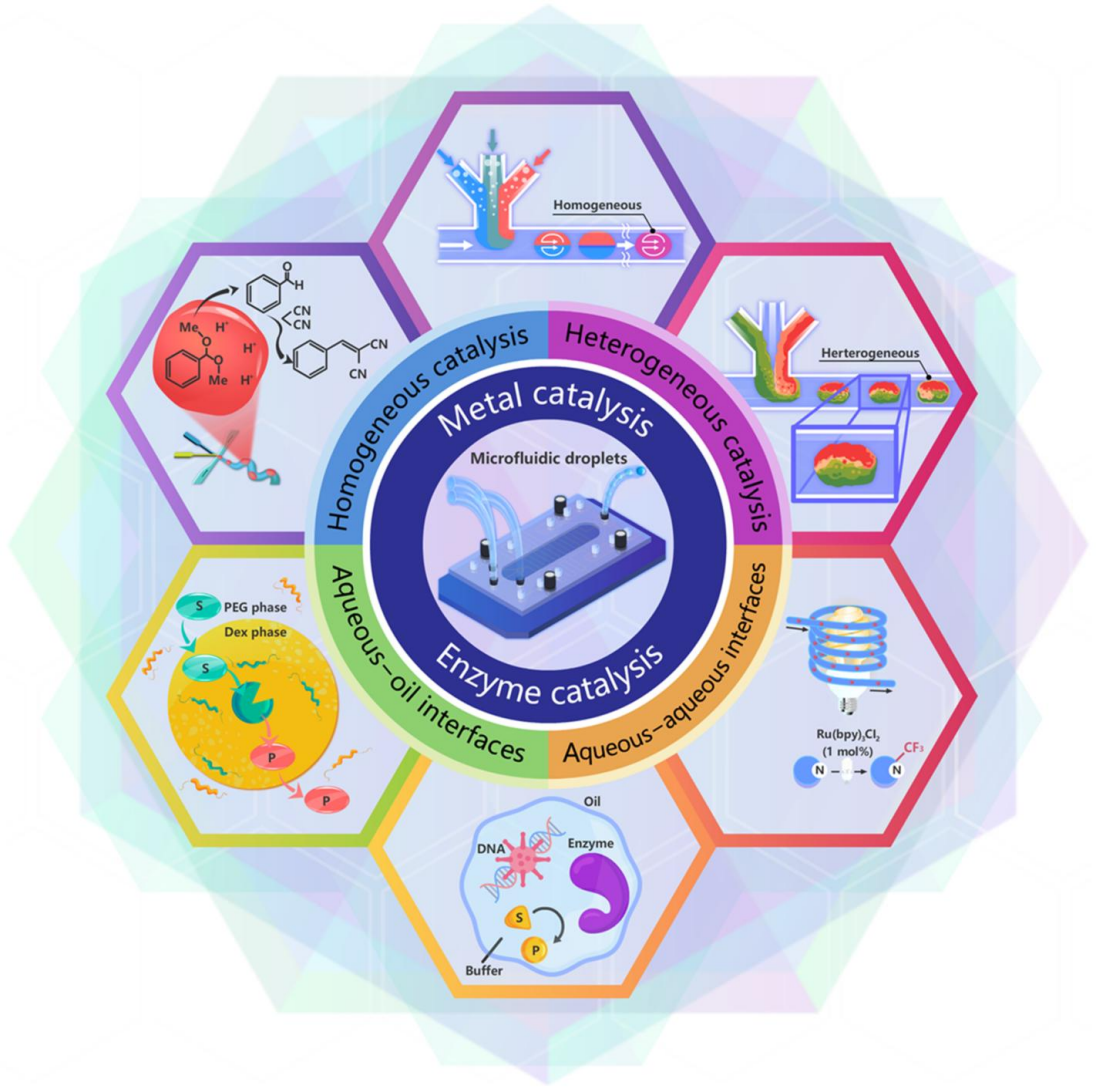
Basic schematic of metal‐based and enzymatic catalyzes in microfluidic droplets.

Therefore, this review presents recent advances in catalytic reactions in microfluidic parameterized droplets. Traditionally, catalysis is divided into three categories: homogeneous catalysis, heterogeneous catalysis, and biocatalysis.^[21]^ Biocatalysis involves the utilization of natural substances, such as enzymes from biological sources or entire cells, to accelerate chemical reactions. For many years, enzymes have been used in a wide range of chemical processes. For instance, millions of tons of acrylamide are produced each year using nitrile hydratases, and detergents have included enzymes for decades.[^22^, ^23^] Thus, this review considers enzyme catalysts as representative biocatalysts. First, the reactions in microdroplets, including homogeneous and heterogeneous catalysis, will be summarized while considering other factors, such as the size of the droplet, pH, temperature, and concentration. Then, how enzymes that catalyze processes in living organisms employ a limited environment, similar to that offered by microdroplets, to achieve a high‐efficiency catalysis activity will be discussed. Finally, the application of droplets in catalysis will be discussed as well as future difficulties and possible applications for these droplet systems (Figure 1).

## 2 HOMOGENEOUS CATALYTIC SYSTEMS

To date, efficient catalysis is still a very active research area. Loading functional catalysts in some microstructures is essential to improving their catalytic activity and selectivity. However, when the supported catalysts come into contact with each other or other materials, they may separate from their carriers.[^24^, ^25^] This issue can be resolved by conducting catalytic reactions in the cavity of microfluidic droplets. The interiors of these droplets can hold catalysts like a container.[^26^, ^27^]

Moreover, the outer layer of microfluidic droplets can serve as a physical and chemical barrier and prevent interactions between different droplets and the catalyst. Additionally, the performance of reactions can be improved by ensuring the free mobility of active catalysts, which can also be guaranteed in microfluidic droplets. Based on the solubility of the catalysts utilized in microfluidic droplets, the catalytic systems have been divided into homogeneous catalytic systems that dissolve well in droplets and poorly dissolved heterogeneous catalytic systems.

As the reaction system only consists of fluid phases that can flow easily in microreactors, homogeneous catalysts are preferable in microfluidic reactions with multiphase catalytic processes. Especially in narrow flow channels, homogeneous catalysts can reduce the possibility of flow choking. Compared with heterogeneous catalysts, switching the conventional homogeneous catalytic system from batch reactors to microfluidic droplet reactors is also quite convenient. The use of microdroplets in homogeneously catalyzed gas‒liquid/liquid‒liquid reaction processes, such as oxidation reactions and other chemical syntheses, has been proven advantageous and promising.[^28^, ^29^, ^30^, ^31^] Hence, the publications discussed in this section will be limited to those involving homogeneous catalysis in microfluidic droplets.

**FIGURE 2:**
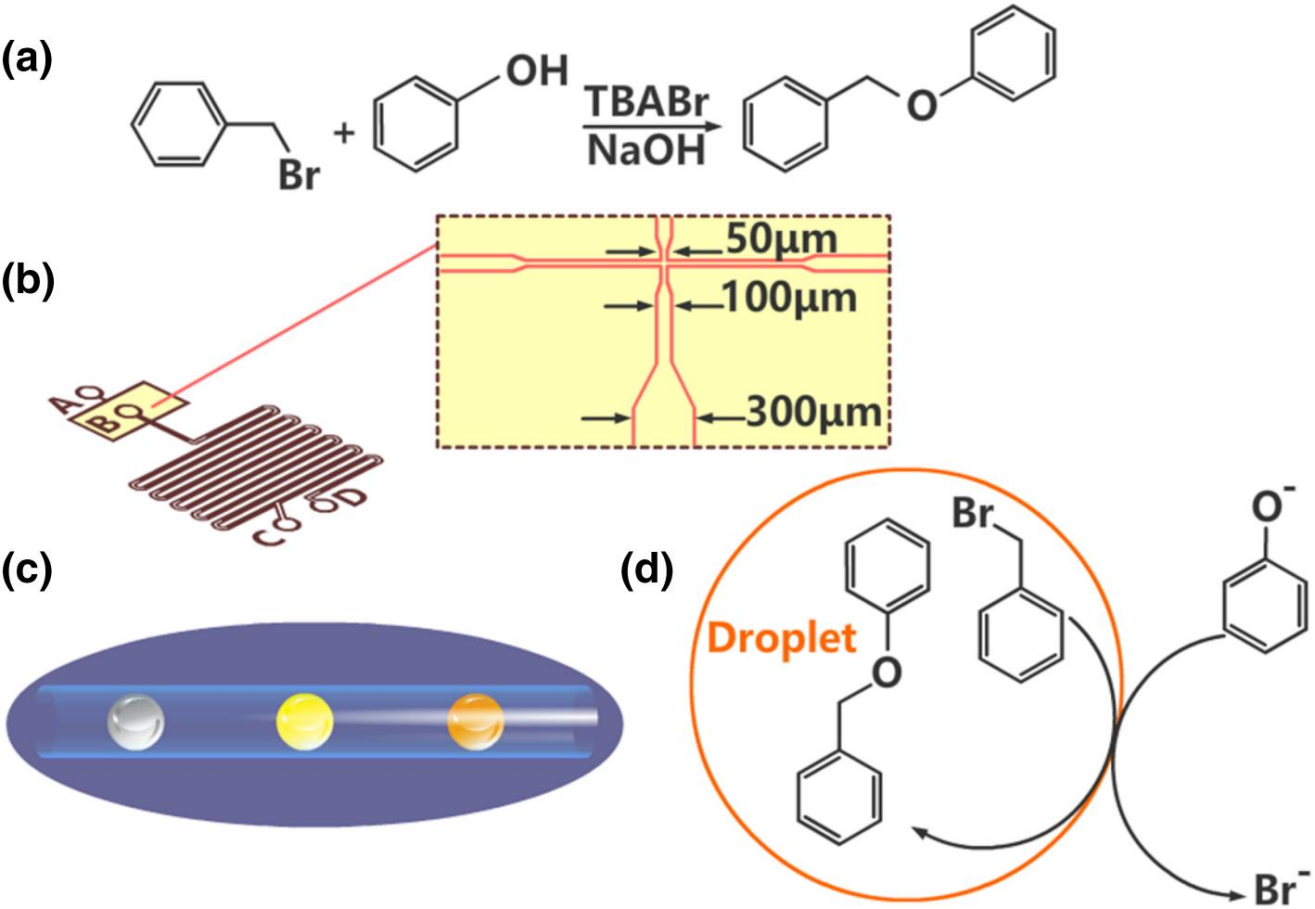
Schematic representations of a droplet‐based interfacial microreactor. (a) Reaction of benzyl bromide and phenol to produce benzyl phenyl ether catalyzed by tetrabutylammonium bromide (TBABr). (b) Overview of the microfluidic chip device. (c) Flow pattern in the microchannel. (d) The phase transfer catalyst TBABr as a surfactant, self‐assembled at the liquid–liquid interface to catalyze the reaction between the aqueous and organic phases while the organic droplets traveled through the hydrophilic microchannels. *Source*: Reproduced from Ref.^[32]^ with permission. Copyright (2012), The Royal Society of Chemistry.

The high surface‐to‐volume ratio and internal flow circulation in microfluidic droplets increase mass transfer and heat transfer, thus accelerating the reaction. For example, the etherification of benzyl bromide and phenoxide using tetrabutylammonium bromide (TBABr) as the catalyst resulted in 95% conversion of phenoxide in a tubular microreactor after only 1 min of residence time as opposed to 180 min in a batch reactor (Figure 2a).^[32]^ The aim is to make use of the microdroplets formed in the channels to facilitate the two‐phase reaction process. To increase the efficiency of liquid–liquid interfacial reactions, the authors fabricated a simple microfluidic device consisting of a flow‐focusing junction as shown in Figure 2b. A spherical liquid–liquid interface was obtained by dispersing one liquid phase into another to form droplets, thus facilitating the two‐phase reactions between immiscible participating fluids (Figure 2c). The TBABr phase transfer catalyst assembled at the droplet “wall” catalyze the reactions between the aqueous and organic phases (Figure 2d). This study illustrates an acceleration approach that is ideal for biphasic reactions by taking advantage of droplet‐based microdevices. Compared to conventional dispersion techniques, this microdevice offers faster interfacial reactions as more effective mass transfer is expected due to the droplets' high surface‐to‐volume ratio and internal flow circulation.

**FIGURE 3:**
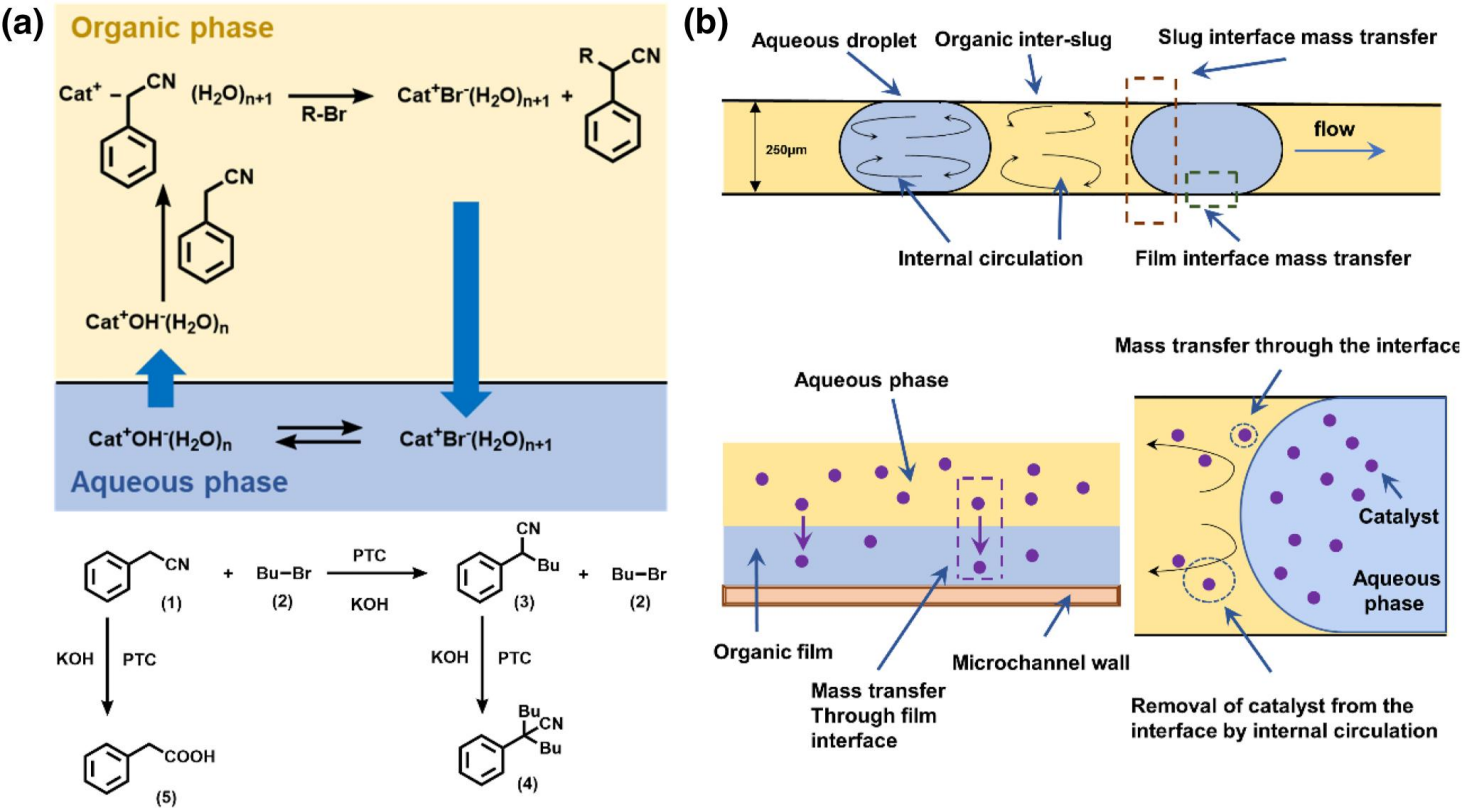
Reactions catalyzed by phase transfer catalysts in microflows. Selective alkylation by a segmented flow reaction in the microchannel. (a) Mass transfer and kinetic steps in the phase‐transfer alkylation of phenylacetonitrile. Cat: triethylbenzylammonium chloride (TEBA). (b) Mass transfer through the interface in a segmented flow. Internal circulation moves the catalyst away from the interface, thus increasing the area of reaction. *Source*: Reproduced from Ref.^[33]^ with permission. Copyright (2010), American Chemical Society.

The precise control of the interfacial area of aqueous and organic droplets in a microchannel reactor is an attractive way to optimize yield and productivity. In another study, microfluidic droplets were used to selectively alkylate phenylacetonitrile with phase transfer catalysts as shown in Figure 3a.^[33]^ The large surface‐to‐volume ratio significantly promoted phase transfer of the catalyst. As the organic droplet size decreased, the degree of internal circulation of the organic liquid in these droplets increased, therefore decreasing the mass‐transfer contact time and increasing the rate of removal of the catalyst and reactant species from the interface, thus further increasing the rate of transport across the interface as shown in Figure 3b. It was evident that smaller droplets boosted the reaction yield by enhancing the mass transfer. The monoalkylated products had significantly higher selectivity and conversion than those produced in a batch reactor.

**FIGURE 4:**
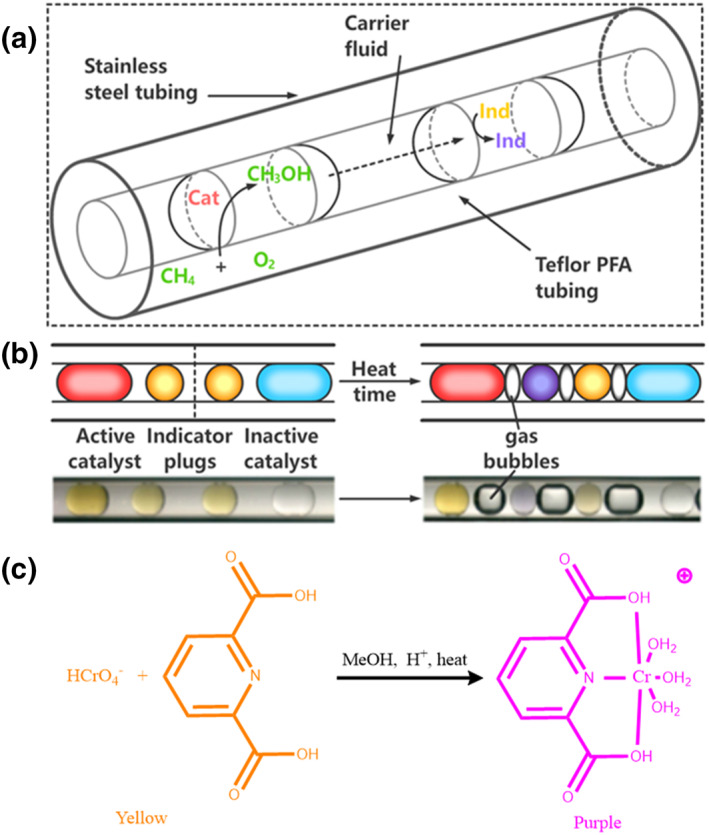
Arrays of microfluidic plugs containing catalyst solutions were reacted with gaseous reagents and then screened for catalytic activity. (a) Schematic of a section of Teflon tubing containing arrays of plugs inside stainless steel tubing. (b) Schematics (above) and microphotographs (below) showing how using two indicator (ind) plugs to separate adjacent catalyst plugs allows clear identification of active catalysts. Only the indicator plugs adjacent to an active catalyst plug changed color. Here, the catalyst plug on the left was active, while the catalyst plug on the right was not. Over time, gas bubbles formed between the plugs due to the pressurization and evaporation of the carrier fluid. (c) Chromic acid oxidized methanol, generating chromium (III), which coordinated to 2,6‐pyridinedicarboxylate, causing a color change from yellow to purple. *Source*: Reproduced from Ref.^[34]^ with permission. Copyright (2010), American Chemical Society.

When too many relevant parameters are available for the traditional optimization process, microfluidic droplet allow quick and effective catalytic parameter optimization. A novel procedure to generate active homogeneous catalyst systems for the aerobic oxidation of methane to methanol was developed by Ismagilov et al. using microfluidic droplets and a genetic algorithm (GA).^[34]^ A GA requires executing a large number of reactions in parallel to optimize high‐fitness catalysts, which was made possible by droplet microfluidics. The small scale and volumes of microfluidic droplets offer significant safety benefits. As shown in Figure 4a, the microfluidic system included methods for creating various arrays of droplets containing catalysts, introducing gaseous reagents at high pressure, carrying out reactions simultaneously, and detecting the catalyst activity using nearby indicator droplets to identify the methanol product in situ. A plug‐based microflow pattern was used to incorporate each distinct catalytic system into an aqueous droplet. A fluorocarbon‐immiscible carrier fluid was employed to separate the water droplets. When methanol was present in the nearby active catalyst droplet, the color of the indicator droplet changed from orange to purple (Figure 4b). The mechanism of action of the indicator is based on the oxidation of the final product methanol by chromic acid (Figure 4c). The success of this protocol proved that GA catalytic system optimization could greatly benefit from the use of microfluidic droplets.

Even though it is convenient to use homogenous catalysts directly dissolved in microfluidic droplets, the inability to recover and reuse these homogeneous catalysts is still an issue that requires further attention. Because of aggregation, the catalysts used in the homogeneous catalysis reaction may suffer from low thermal stability. The separation of homogeneous catalysts from the generated products is also a challenging issue that causes product contamination. Thus, distillation is required to separate the organic products from the homogeneous catalysts. One of the main benefits of using a heterogeneous catalyst is that it can be straightforward to separate it from a reaction mixture, for example, via filtration. Using heterogeneous catalysts instead of homogeneous catalysts could potentially lead to lower production costs by enabling the reuse of the catalysts and simplifying the purification procedure. Therefore, expensive catalysts could be easily and effectively recovered, which is an important consideration, especially for industrial‐scale manufacturing processes.

## 3 HETEROGENEOUS CATALYTIC SYSTEMS

**FIGURE 5:**
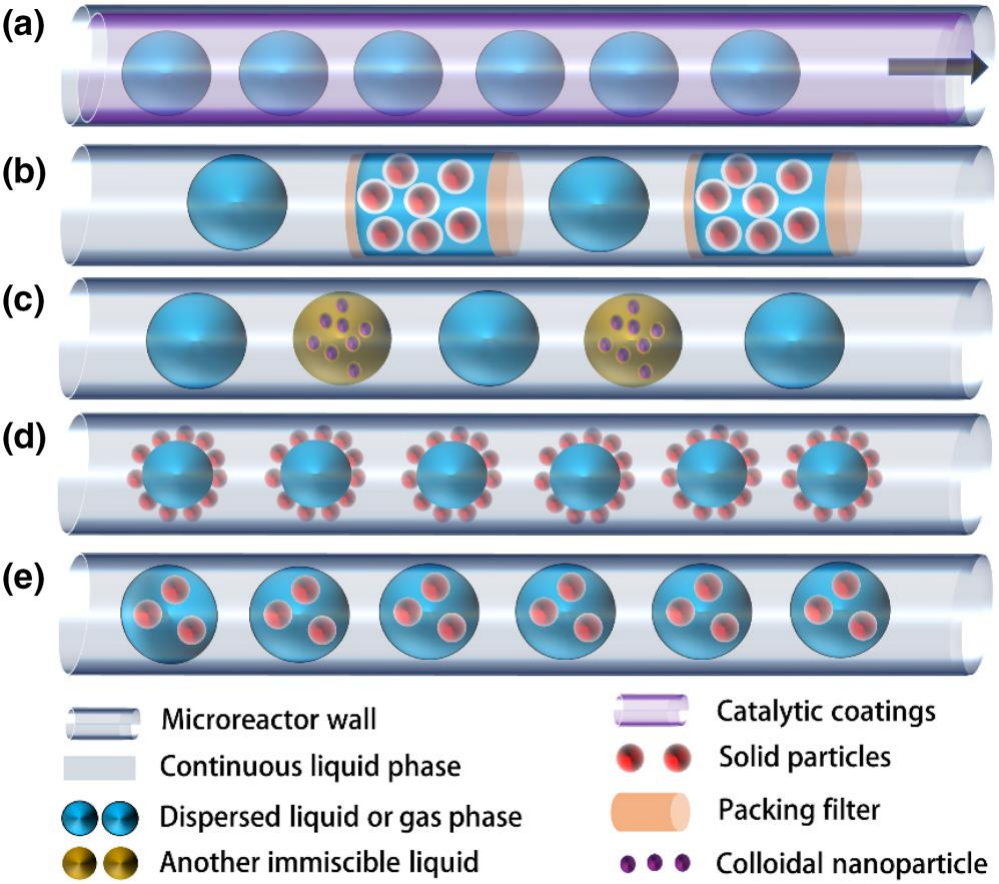
Examples of multiphase reactions in microreactors with (a) wall‐coated catalysts, (b) packed bed catalysts, colloidal nanoparticle suspension catalysts with (c) gas bubbles, (d) PEs, and (e) slurry catalysts. *Source*: Reproduced from Ref.^[19]^ with permission. Copyright (2022), American Chemical Society. PE, Pickering emulsion.

Heterogeneous catalysts are more attractive than homogeneous catalysts in terms of catalyst separation and recycling in microreactors. Traditionally, solid catalysts are fixed in microreactors either by being coated on a wall (Figure 5a) or by being packed in a bed (Figure 5b).^[19]^ Recently, the continuous transport of solid catalysts in flows has enabled flexible and multipurpose production and has become a viable alternative for carrying out heterogeneously catalyzed reactions in microreactors.^[35]^ The distribution of solids within the liquid flow can be controlled by adjusting the particle–fluid and particle–surface interactions.^[36]^ Several solid suspension systems in microreactors have been reported based on these interaction differences. For example, colloids with a suspension of nanoparticles are typically micrometer‐sized solid particle suspension systems in microreactors (Figure 5c). In addition, Pickering emulsions (PEs) contain solid particles of nanometer or micrometer size stabilized at the two immiscible liquid‒liquid interfaces (Figure 5d). Finally, slurries are another typical micrometer‐sized solid particle suspension system found in microreactors (Figure 5e).^[19]^

Compared with that in conventional batch reactors, incorporating catalysts in microfluidic droplets opens a new avenue for realizing efficient heterogeneous catalysis and significant process intensification, which is thus the topic of this section. The current knowledge has been summarized to elucidate the effects of diverse catalyst forms, including wall‐coated, packed bed, colloid, PE, and slurry catalysts, on the microflow characteristics and catalytic reaction enhancement in microdroplets, followed by a discussion of the future challenges and a perspective on the potential applications of such microfluidic droplet systems.

### 3.1 Wall‐coated catalysts

A typical way to incorporate solid catalysts into droplet microfluidics is to cover the inner wall of the microreactor with a thin layer of catalytic coating. The uniform deposition of a catalytic layer around microreactor walls usually results in a thickness in the order of 1–10 μm.[^37^, ^38^] Wall coating is an attractive approach for immobilizing catalysts into microchannels due to the potential enhancement of heat and mass transfer.^[39]^ Heterogeneously catalyzed gas‒liquid hydrogenation reactions are commonly constrained by mass transfer between the liquid and solid phases. Additionally, wall coating has the advantages of a well‐defined flow pattern generated in empty microchannels and the lack of the high pressure drop that is generated in packed bed microreactors (vide infra).

**FIGURE 6:**
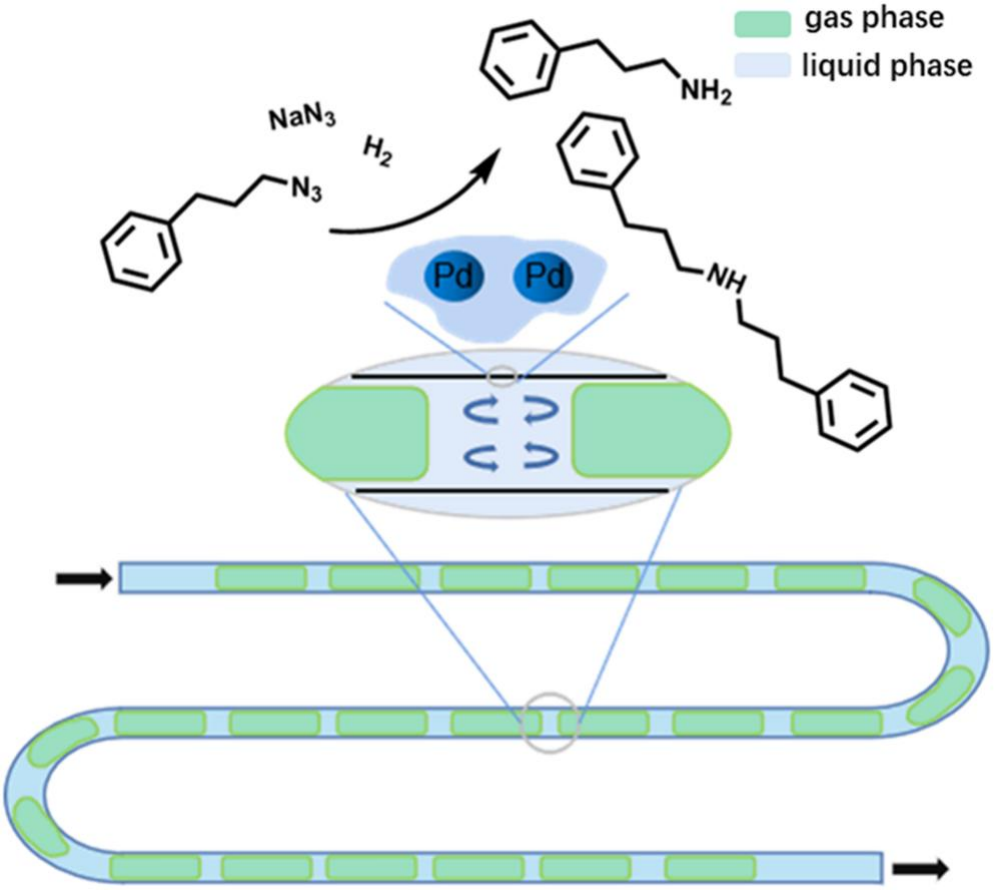
Continuous capillary microreactor with a Pd catalyst immobilized on the inner wall operated under segmented gas–liquid flow. *Source*: Reproduced from Ref.^[40]^ with permission. Copyright (2011), John Wiley and Sons.

Most studies in this area have focused on immiscible catalysts, involving reactions, such as gas‒liquid hydrogenation, aerobic alcohol oxidation, the direct combination of hydrogen and oxygen for hydrogen peroxide synthesis, and epoxidation with aqueous H_2_O_2_ solutions. For these reactions, interphase mass transfer commonly restricts the total reaction rate in conventional reactors when the reaction kinetics are rapid. The mass transfer rate is significantly increased when such multiphase reactions are carried out over a thin catalyst layer coated in microreactors. This, plus unique reaction parameter control in microfluidic droplet systems can lead to a much‐improved reaction performance. Kreutzer et al. performed the hydrogenation of 3‐methyl‐1‐pentyn‐3‐ol and generated the desired 3‐methyl‐1‐penten‐3‐ol in a fused silica capillary microreactor coated with 0.003–5.7 wt % Pd/γ‐Al_2_O_3_ catalysts (Figure 6).^[40]^ It was demonstrated that a rapid mass transfer rate in microfluidic droplet systems might overcome the transport limitation, enabling the reaction to proceed under intrinsic kinetic control. Additionally, a 78% yield of the final product of 3‐methyl‐1‐penten‐3‐ol was achieved within 1 min of residence time. The catalytic coating showed no signs of deactivation over several weeks. Combined with microfluidic droplets, the catalytic coating with long‐term stability has industrial application potential.

### 3.2 Packed bed catalysts

In a packed bed catalyst, a certain length of the microreactor channel is filled with solid catalysts so that catalyst movement is restricted. In a packed bed catalyst in droplet microfluidics, the reactants are forced into contact with the immobilized catalyst. Furthermore, the confinement of the heterogeneous catalysts within the reactor leads to a high relative concentration of the catalyst in comparison to that of the flowing reagents in the microfluidic droplets, which significantly boosts the catalytic efficiency. Because the catalyst is stationary, the loss of catalysts due to attrition is decreased, and the lifespan of the catalysts is increased.^[41]^

**FIGURE 7:**
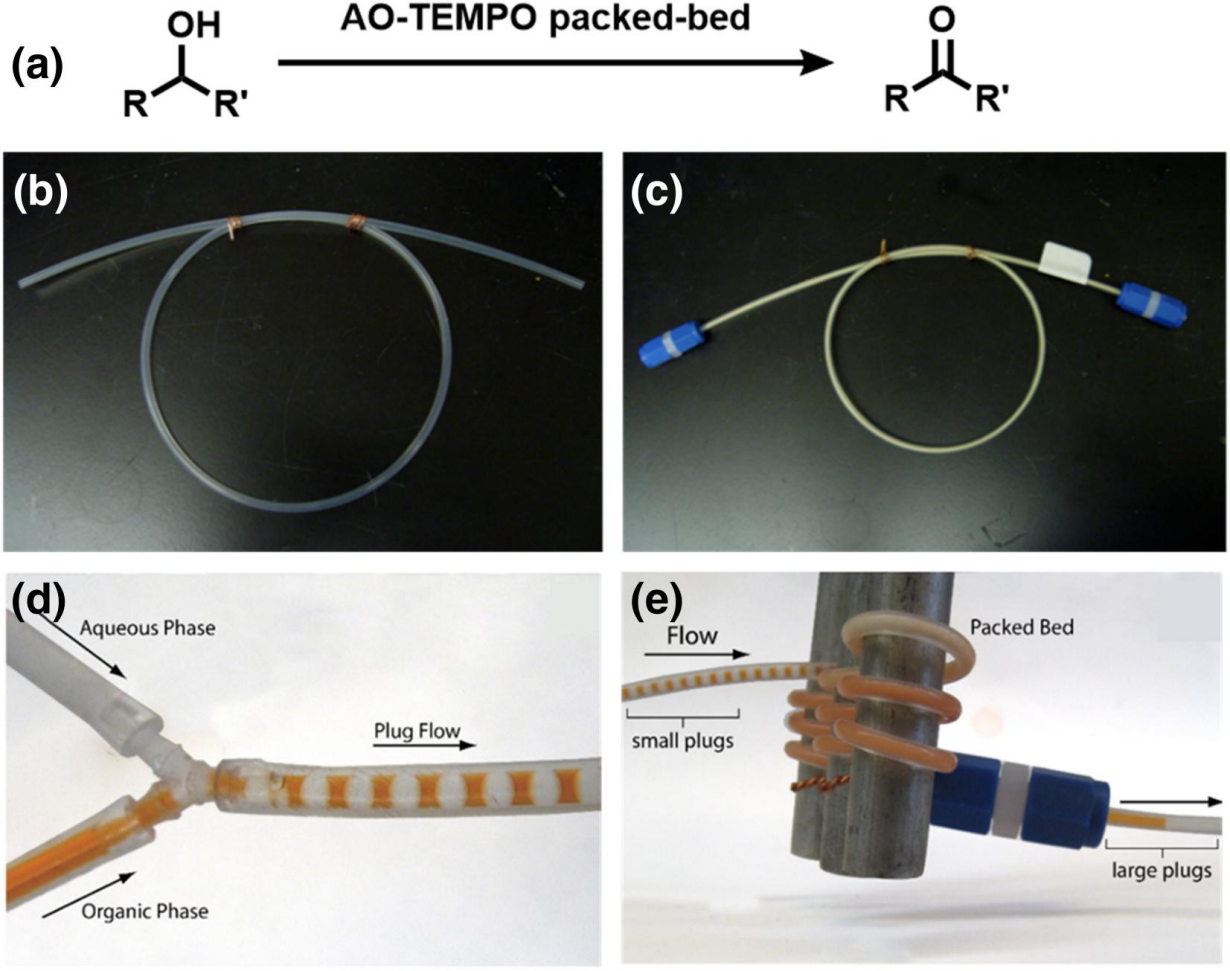
(a) Oxidation of alcohols using an AMBERZYME® oxirane‐tetramethylpiperidine‐1‐oxyl (AO‐TEMPO) packed‐bed microreactor. (b) An empty microreactor channel. (c) Microreactor channel packed with functionalized AO resin and attached to caps. (d) Organic (colored solution) and aqueous (colorless solution) phases forming plugs at the Y‐junction. (e) Phases mixing upon reaching the packed bed, leading to a coalescence of drops at the outlet of the microchannel.^[42]^

In capillary or chip‐based microreactors, solid catalysts can be directly packed with powder particles. This simple and practical method of catalyst integration allows the direct use of commercial or lab‐made bulk catalysts and thus greatly expands the applications of microreactors in solid‐catalyzed multiphase reactions. Liquid flow within this type of bed is generally plug flow but can be turbulent at higher flow rates. McQuade et al. described a packed‐bed microreactor that uses an immobilized tetramethylpiperidine‐1‐oxyl (TEMPO) catalyst to oxidize primary and secondary alcohols in a plug droplet flow pattern (Figure 7a).^[42]^ Figure 7b,c show that the microreactor channel is packed with functionalized AMBERZYME® oxirane (AO) resins and is attached to caps. Although organic alcohols and aqueous oxidants enter the reactor as plugs (Figure 7d), they combine to form an emulsion on the packed bed. After leaving the reactor, the emulsion solidifies into larger plugs, keeping the organic product and the watery byproducts separated (Figure 7e). Oxidation occurs with high yields and excellent stability over time. In addition, the microreactor can be used to successfully oxidize a variety of alcohols and can continue to function after more than 100 trials without losing any catalytic activity. This means that the packed bed catalysts made from AMBERZYME® oxirane resins with immobilized TEMPO (AO‐TEMPO) are recyclable and can maintain their catalytic activity over time in droplet microfluidic systems.

### 3.3 Colloidal nanoparticle suspensions in microfluidic droplets

**FIGURE 8:**
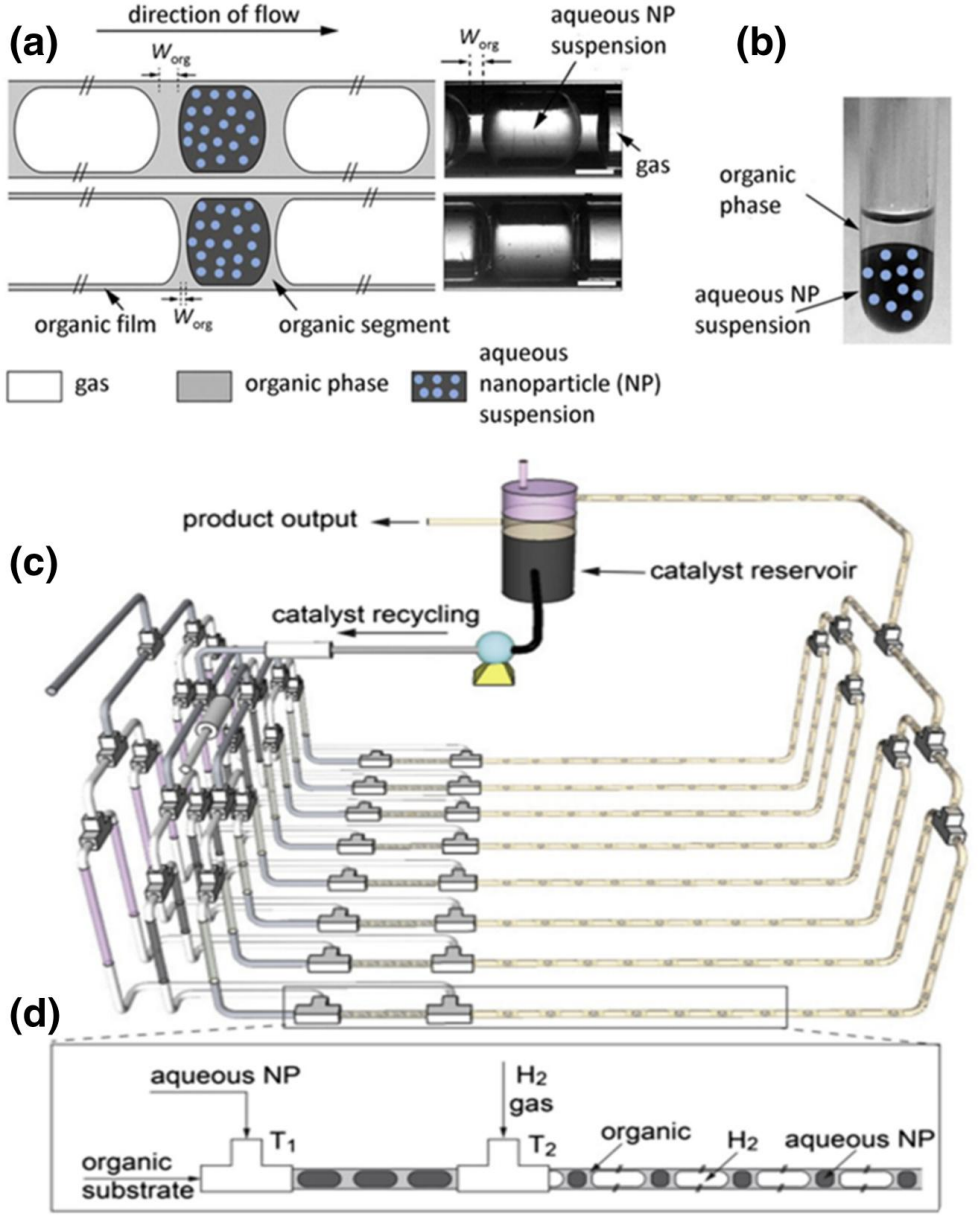
(a) Schematic of triphasic slug flow. (b) Catalyst recovery from aqueous‐organic phase separation. (c) Schematic of the triphasic flow reactor system for continuous catalyst recycling in the PtNP‐catalyzed hydrogenation of nitrobenzene. (d) The formation of triphasic flow in each reactor. *Source*: Panels (a) and (b) were reproduced from Ref.^[43]^. Copyright (2014), The Royal Society of Chemistry. *Source*: Parts (c) and (d) were reproduced from Ref.[^19^, ^44^] with permission. Copyright (2022), American Chemical Society and Copyright (2016), Elsevier Science Direct.

Colloidal suspensions of nanoparticles are heterogeneous mixtures with significant flow mobility rather than genuine solutions. A typical colloidal nanoparticle suspension in microfluidic droplets is presented in Figure 8a.^[43]^ Colloidal catalysts exhibit strong catalytic activity compared to homogeneous catalysts due to their nanometer‐scale particle size and have heterogeneous catalytic characteristics that make catalyst separation and recovery very simple. Simple phase separation by decantation allows the facile recovery and reuse of colloidal catalysts in the aqueous phase (Figure 8b).[^45^, ^46^] They have gained widespread attention in catalytic applications and have slowly progressed into the field of microfluidic droplets to achieve significant catalytic performance improvement.[^47^, ^48^]

A good dispersion in droplets is needed for colloidal nanoparticles to be widely used in droplet microfluidics. Several traditional methods, including dispersant addition, magnetic stirring, and sonication, have been used to improve the dispersion of nanoparticles in the prepared solution. Utilizing dispersants or surfactants, which can reduce the surface tension of the base fluid and prevent nanoparticle aggregation, is the most economical technique for boosting the stability of colloids. Due to their high mobility, colloidal nanoparticle suspensions behave like pseudohomogeneous liquid phases and flow‐like pure liquids. As a result, multiphase flow patterns involving colloidal nanoparticle suspensions appear similar to those involving homogeneous pure liquids.

This pseudohomogeneous characteristic offers robust reactivity in microfluidic droplets and facile scale up. For instance, Khan et al.^[44]^ reported a colloidal catalyst‐catalyzed hydrogenation of nitrobenzene with a robust new eightfold parallelized three‐phase segmented‐flow reactor network at an approximately constant substrate conversion of 80% under ambient conditions with continuous online recovery and recycling of the colloidal catalyst phase over 5 h of operation (Figure 8c). An increase in reaction productivity was achieved via these parallelized microreactor networks integrated with online catalyst recycling in which the desired flow pattern for rapid mass transfer, as depicted in Figure 8d, was still maintained. This model sheds new light on the robust reactivity of microfluidic droplets and important parallelized microfluidic reactor networks for stable long‐term operation.

### 3.4 PEs in microreactors

A PE is a type of emulsion that is kept stable by solid particles that adhere to a liquid‒liquid interface formed by two immiscible liquids. The size of the solid particles is between 10 nm and 100 μm, while the PE drops typically range in size from 10 to 1000 μm.^[49]^ PEs have attracted much attention because of their applications in the food, pharmaceutical, and biomedical industries. In particular, they are useful for biphasic catalysis.^[50]^ Thus, PEs offer another possibility for enhancing the reaction efficiency in microfluidic droplets.^[51]^

It is desirable for future synthetic chemistry to mimic living systems to process multistep cascade reactions in a one‐pot fashion. One of the critical challenges is the mutual destruction of incompatible or opposing reagents, for example, acids and bases or oxidants and reductants. In particular, compared with a traditional biphasic droplet microfluidic system, a PE system enables reactions to be carried out with incompatible reagents by separating the opposing reactants in different compartments. This procedure consists of preparing two parent water‐in‐oil (w/o) PEs, each containing one of the incompatible reagents.

**FIGURE 9:**
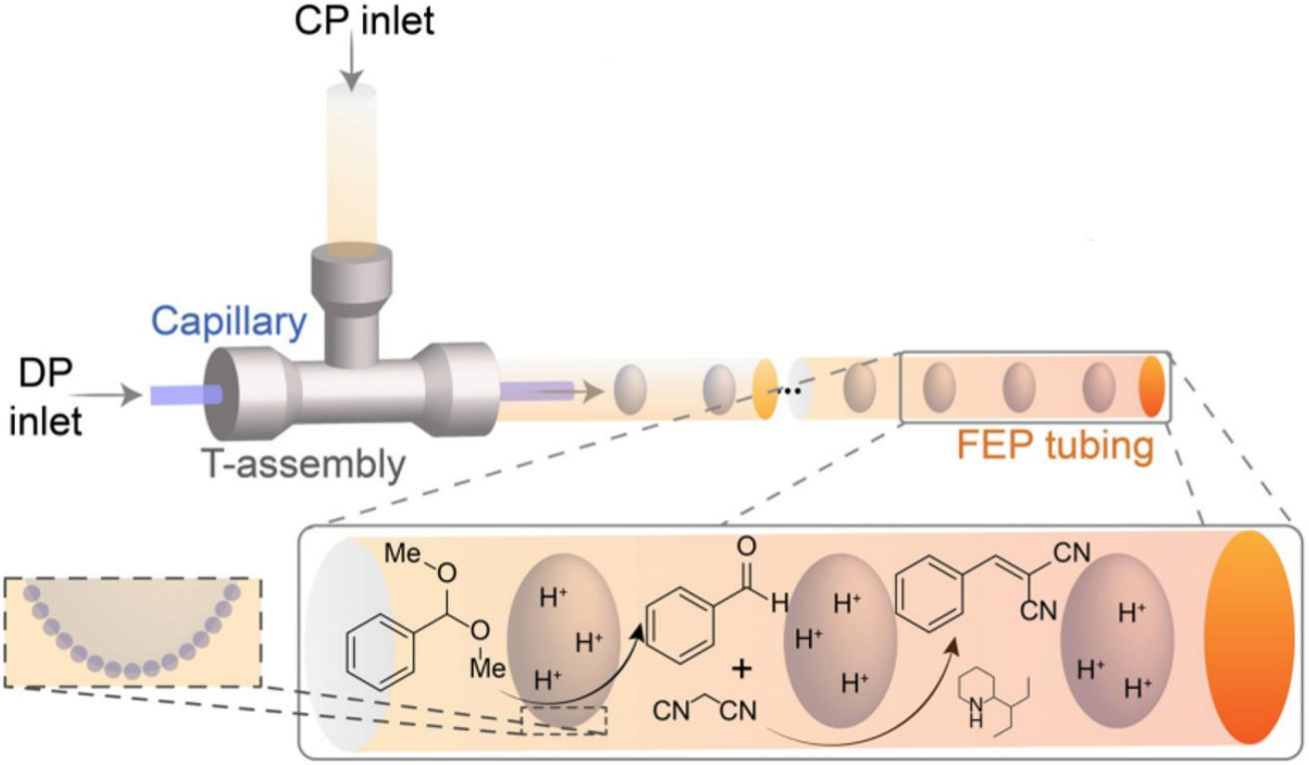
Droplet microfluidic approach for in‐flow PE catalysis with 4‐propylguaiacol as the continuous phase and water as the dispersed phase. Bottom inset: Scheme of the deacetalization–Knoevenagel reaction inside the tubing, including a depiction of the particles on the droplet surface. *Source*: Reproduced from Ref.^[51]^ with permission. Copyright (2020), John Wiley and Sons. PE, Pickering emulsion.

By applying droplet microfluidics, Bruijnincx et al. carried out a deacetalization‐Knoevenagel condensation in PEs stabilized with partially hydrophobic silica nanospheres, where HCl and the base 2‐(1‐ethylpropyl)piperidine catalyzed deacetalization and Knoevenagel condensation, respectively (Figure 9). In principle, it is impossible to combine these two reactions in a single vessel as HCl and 2‐(1‐ethylpropyl)piperidine rapidly react with each other. Due to rapid acid‐base quenching, the reaction yield is very low (yielding 10% and 17% of the intermediate benzaldehyde and the final product benzylidene malononitrile, respectively) in a traditional biphasic flow system. Because of the physical boundary present at the interface between the two phases in PE droplets, acid‐base quenching occurs much more slowly in this system, resulting in a significantly improved yield (yielding 69% benzaldehyde and 25% of the final product benzylidene malononitrile). In addition, PEs can separate solid particles from the reaction mixture by centrifugation alone, making postprocessing much simpler. An important potential application of PEs in droplet microfluidics is to use them as a vehicle to carry out multiple consecutive reactions with incompatible reagents. This has the advantages of avoiding the purification and isolation of intermediates and saving time, energy, and resources.

### 3.5 Slurry catalysts in microreactors

Slurry catalysts are prepared by suspending microscale catalyst particles in a mobile fluid phase with a typical solid content below 25 mg/mL. They have been used in a variety of catalytic reactions, including hydrogenation, oxidation, fluorination, polymerization, and reduction reactions. For commonly used wall‐coated and packed bed catalysts in microfluidics, removing the catalysts without endangering the reactor is difficult, particularly for commercial applications.^[37]^ To address the issues associated with wall‐coated and packed bed catalysts, slurry catalysts for microfluidic droplets were created. Slurry catalysts are far simpler to fabricate and much less expensive than wall‐coated and packed bed catalysts. Compared to packed bed catalysts, slurry catalysts have other significant advantages, such as a low pressure drop, a large catalyst area, easy removal of the catalyst, and low catalyst consumption.

**FIGURE 10:**
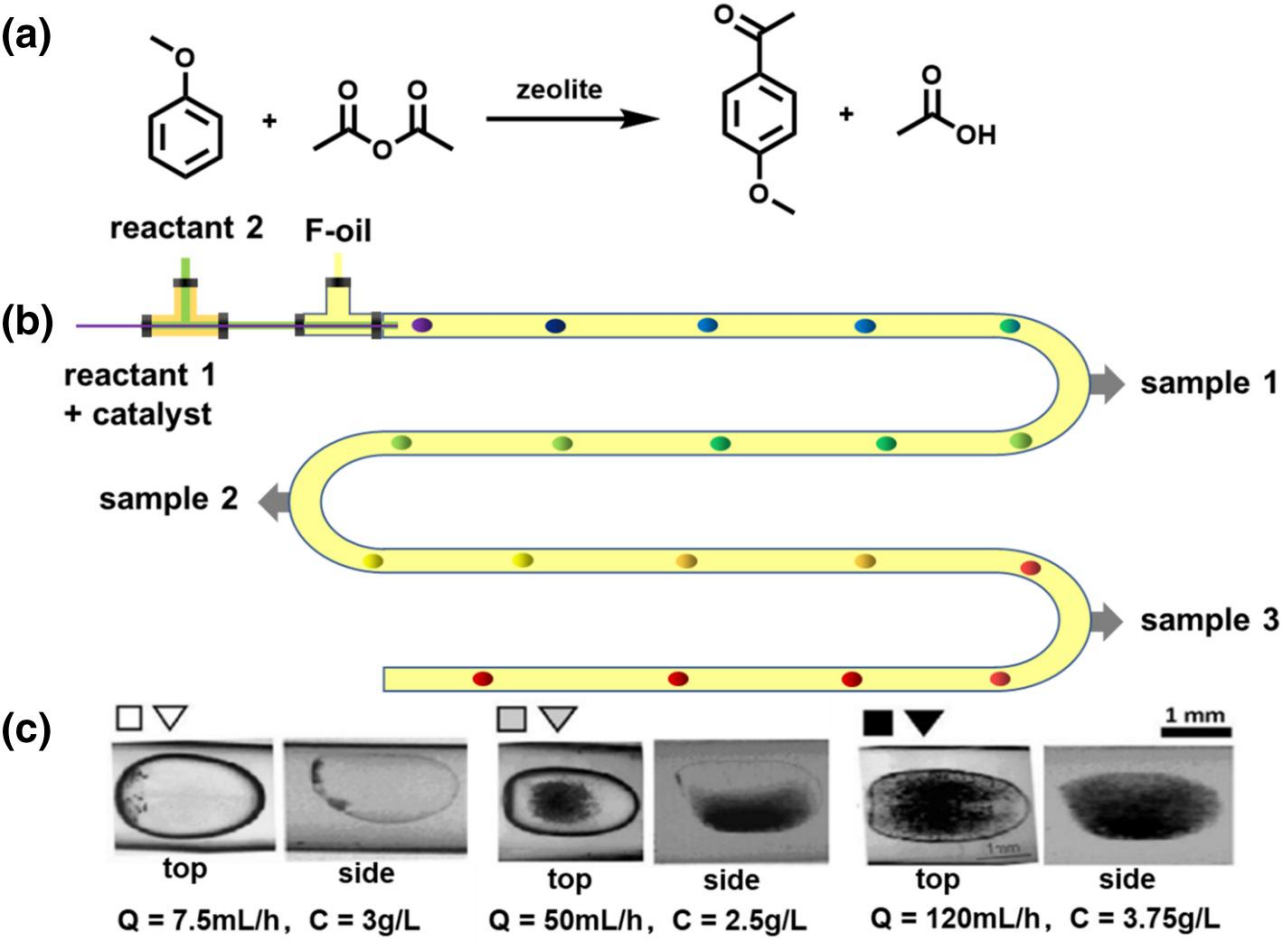
(a) Anisole acylation catalyzed by zeolites. (b) Schematic view of the millifluidic device used for liquid–liquid–solid flow. (c) In the dispersed ethanol phase (fluorinated oil as the continuous phase) at different total flow rates (Q) and solid loadings (C). *Source*: Reproduced from Ref.^[52]^ with permission. Copyright (2013), Elsevier Science Direct.

Additionally, because the slurry phase is thoroughly mixed, it offers the benefit of rapid heat evacuation and a more stable reactor temperature with the elimination of local hot spots. In this way, much higher temperatures on average can be achieved without the danger of sintering the catalyst. These advantages led to the investigation of possible anisole acylation over slurry catalysts in a microfluidic droplet at a high temperature of 90–120°C (Figure 10a).^[52]^ Microfluidic droplets formed at a concentric junction where the reactants met an inert carrier phase as shown in the schematic in Figure 10b. Because of the use of a fast camera, droplet flow patterns with the slurry solid catalysts inside the drops were captured as shown in Figure 10c. This method offers a droplet‐flow device with the use of a minute amount of solid heterogeneous catalysts for catalyst screening. Although high‐throughput catalyst screening is the main emphasis of this research paper, it also provides significant insight into solid flow manipulation for improving the efficiency of liquid‒solid interactions under suspension catalysis in droplet microfluidics.

## 4 OUTLOOK OF HOMOGENEOUS AND HETEROGENEOUS CATALYTIC SYSTEMS IN MICROFLUIDIC DROPLETS: CHALLENGES AND OPPORTUNITIES

Droplet microfluidics offers excellent opportunities to efficiently carry out catalytic reactions in flows by loading solid catalysts with diverse protocols. Catalyst particles in droplet microfluidics facilitate flexible manufacturing in a reaction environment similar to that of cells and, moreover, provide a good or even better heat/mass transfer rate than classic microfluidic methods.^[53]^ Droplet microfluidics has exhibited promising reaction applications using wall‐coated, packed bed, colloidal suspension, PE, and slurry catalysts. The enhanced transfer under such particle‐laden flow processing in droplet microfluidics allows the attainment of a maximum reaction rate or superior performance in terms of the reaction conversion or product yield compared with that under their batch counterparts. Because the time needed to achieve miscibility and diffusion of the reactant molecules is drastically reduced in droplet microfluidic reactors compared to that needed in traditional batch reactors, droplet microfluidic reactors have reduced erosion issues, and less energy is required per unit of reactants for mixing and heating.

In droplet microfluidics, various loading techniques for catalysts enable one to test many approaches to identify the most effective ones. Although microfluidic droplets exhibit significant application benefits, there are still several obstacles to be addressed before they may be successfully applied in industrial practice. There is no perfect droplet microfluidic protocol because each one has drawbacks. In general, heterogeneous catalysts are less problematic than homogenous catalysts. However, there are still certain drawbacks related to their use, such as their high cost and efficiency loss after numerous cycles of reuse. Most droplet microfluidics that exhibit a low‐efficiency loss for catalyst reuse cycles are expensive and complex, which cause problems during scaling up. It is necessary to increase the stability of catalysts during flow processing in droplet microfluidic microreactors under reaction circumstances. Creating more straightforward processes for the simple recovery of catalysts in flows and their subsequent reuse without performance degradation is also necessary. To increase production capacity, scaling‐up tactics in droplet microfluidics that consider local particle hydrodynamics must be investigated. There are many potential applications for utilizing microfluidic droplets with solid catalyst particles in various multiphase catalytic reactions, which are still a very understudied field. The hydrodynamics and mass transfer of these solid‐laden droplet microfluidics also require extensive further study to better understand their performance and realize their full potential for enhancing chemical processes.

Due to the requirement for automated chemical manufacturing that decreases production costs and improves the quality of final products, there has been a recent increase in interest in applying machine learning (ML) to guide high‐throughput approaches and high‐quality industrial production. Although there has been much research on the application of ML in high‐throughput screening in experimental research, there have been relatively few studies on ML models for the control, optimization, and real‐time monitoring of industrial systems in the real world. Therefore, future research should concentrate more effort on the combination of droplet microfluidics and ML for large‐scale production facility control, monitoring, and optimization. Future research should also examine the sustainability aspects of droplet microfluidics utilizing cutting‐edge sustainability assessment methodologies, including life cycle assessment, exergy, and their combinations. Based on the discussion above, it can be assumed that droplet microfluidics has the potential to considerably increase the range of applications for microfluidic techniques to accelerate a number of chemical industry reactions and to open the door for novel catalysts.

## 5 ENZYME CATALYSIS

Similar to metal‐based homogeneous and heterogeneous catalysts, biological enzymes require an interface and a water phase for catalysis to occur. As a result, we divided the catalysis of biological enzymes into two types: water‐in‐water (w/w) and w/o. The purposes of this section are to review the important progress in biological enzyme catalysis in droplets, discuss the existing problems and future development directions, and promote the development of high‐throughput enzyme screening techniques and directed evolution methods.

Enzyme catalysis differs from traditional metal‐ and small molecule‐based catalysis in its absolute specificity. The selectivity of enzyme catalysis depends on binding to specific sites of the substrate. An enzyme‐catalyzed reaction presents cascaded amplification under a specific stirring time and environment of the solution, such as the polymerase chain reaction. The enzymatic catalysis that occurs in organisms is often restricted to the internal environment of cells, bacteria, or viruses, which differs greatly from the environment of open solutions. Although enzymatic catalysts have received much attention in the chemical synthesis, their development has been relatively slow compared with that of other catalytic reactions. In addition to having a structure similar to that of cells, drops can simulate a real cellular environment through changes in composition, making them a good choice for studying biological enzyme‐catalyzed reactions.[^54^, ^55^, ^56^, ^57^]

Microfluidic droplets have unique chemical properties that are analogous to test tubes or microplates, where the signals from many droplets are averaged to provide higher sensitivity and detection limits.[^58^, ^59^, ^60^, ^61^, ^62^] Microfluidic technology also has the following advantages^[63]^: (1) significantly reduced sample/reagent volume and ultrahigh analytical throughput^[64]^; (2) precise control of heat and mass transport^[65]^; (3) different functional modules can be easily integrated into microfluidic instruments^[66]^; (4) rapid temperature equilibration and mixing of reactants with efficient heat and mass transfer^[67]^; and (5) extraction of the thermodynamic and kinetic information of enzymes through the high‐throughput extraction of reactants, allowing unbiased analysis and analysis of the reactants, which allows the thermodynamic study of fragile enzymes.^[68]^ In this section, we review the organic synthesis processes enabled by biological enzymes in microfluidics, including homogeneous w/w interfaces and heterogeneous w/o interfaces.

### 5.1 Enzyme catalysis at w/w interfaces

Because of their high biocompatibility, w/w emulsions are particularly useful for biomedical applications, such as the encapsulation of cells, bioreactors, and biomacromolecule processing. Such w/w emulsions are formed by spontaneous phase separation between liquid phases due to the thermodynamic instability of these phases. As a result of ultralow interfacial tension, the generation of liquid‒liquid phases and the stabilization of uniform w/w droplets are challenging. Consequently, it is crucial to design and develop methods for creating w/w droplets for enzyme catalysis.

**FIGURE 11:**
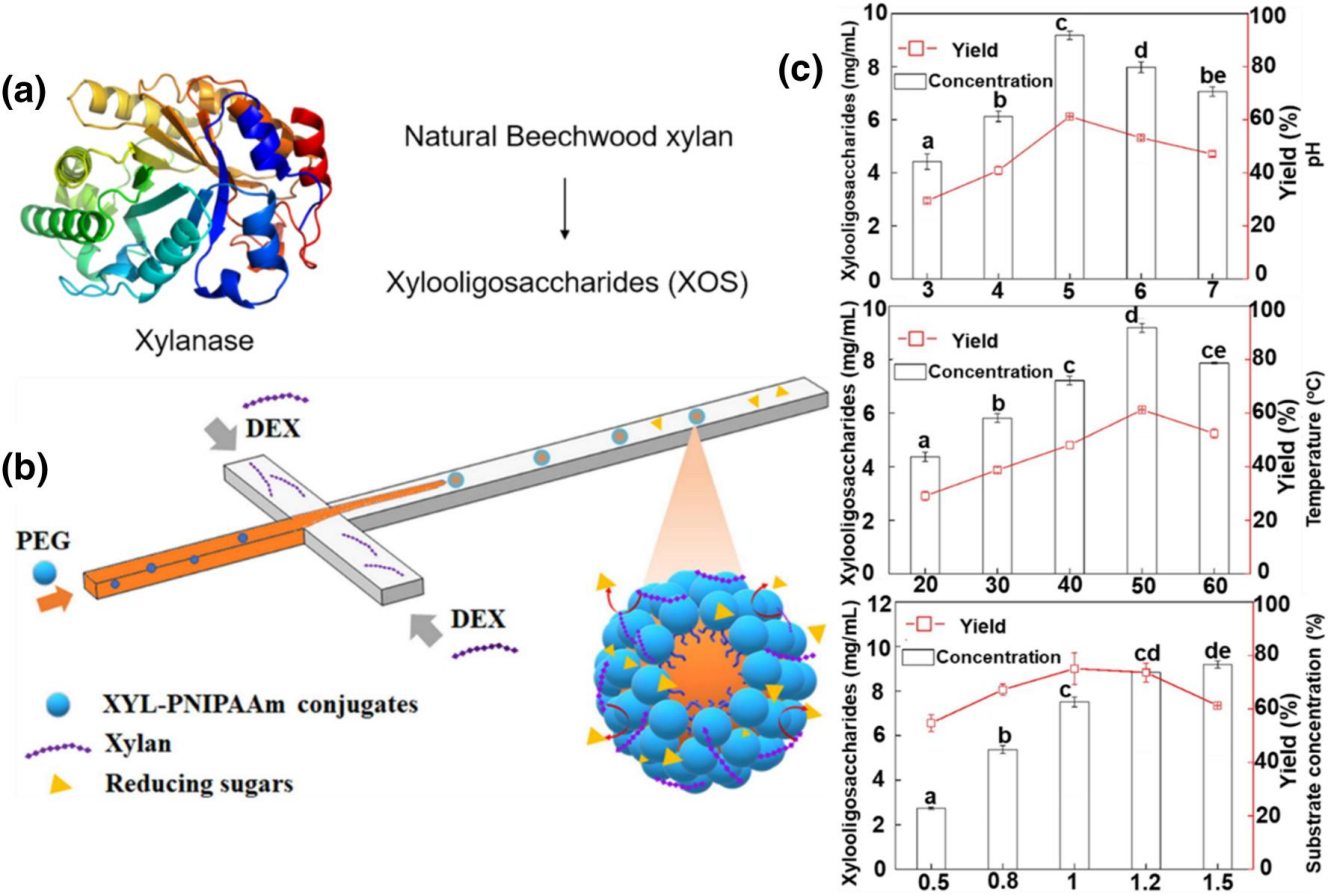
(a) Enzyme‐catalyzed reaction process. (b) Schematic of a w/w droplet‐based microfluidic system for the formation of XOS via the polymerization of Dex with the use of polymerase. (c) Yield of XOS at different pH values, temperatures, and substrate concentrations. *Source*: Reproduced from Ref.^[69]^ with permission. Copyright (2022), Elsevier Science Direct. PEG, polyethylene glycol.

Immobilizing enzymes in w/w emulsion systems is a promising way to increase their stability and catalytic performance. Furthermore, microfluidics has been found to be advantageous in generating large amounts of droplets rapidly and efficiently and provides an effective system for studying the optimal conditions for enzymatic reactions in different environments. As a type of biological enzymes, ligases form chemical bonds between two fragments to form new substances. Thus, understanding how ligases in droplets catalyze reactions is crucial to achieving the compound synthesis. Zhang et al. reported the polymerization of dextran (Dex) into xylooligosaccharides (XOS) with lower molecular weight using polymerase enzymes (Figure 11a) in droplets.^[69]^ Xylanase‐polymer conjugates were prepared first, and then w/w droplets were formed on them. In microfluidic systems, polyethylene glycol (PEG) and Dex were used as the dispersed and continuous phases, respectively (Figure 11b). Regulating the flow rates of the dispersed and continuous phases to control the size of the droplets is simple. Mass and heat transfer are more efficient in droplets because of their large specific surface areas. In the droplet state, the enzyme is in closer contact with the substrate, resulting in a very high rate of conversion at lower concentrations of xylan. This microfluidics approach allows high‐throughput enzyme‐catalyzed reactions to be performed at a variety of pH values, temperatures, and substrate concentrations and allows the optimal conditions for enzyme catalysis to be determined (Figure 11c). In this study, it is demonstrated that the introduction of immobilized enzymes into microfluidic continuous‐flow droplets can significantly improve the catalytic efficiency, providing a general solution for enzyme catalysis in microfluidic droplets.

**FIGURE 12:**
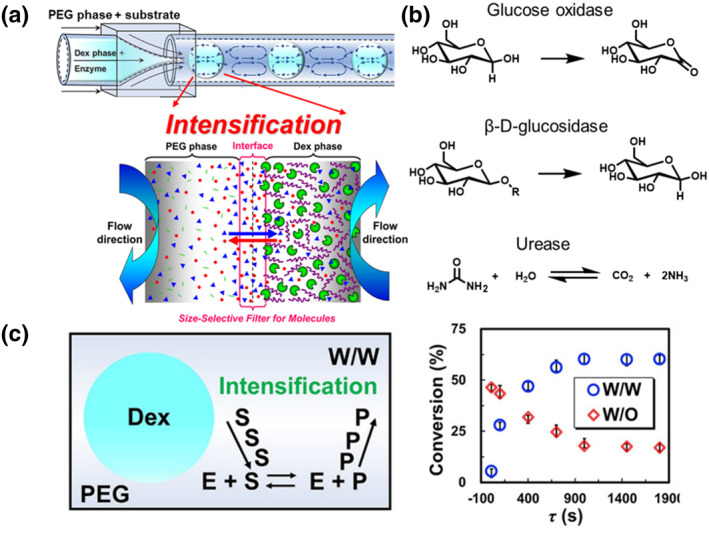
(a) Schematic of an aqueous two‐phase droplet bioreactor. As the substrate diffuses into the bioreactor, it is susceptible to reacting with the enzyme inside the droplet. Selectively permeable interfaces formed from the polyethylene glycol (PEG) and Dex phases allow substrates and products to be transported freely. (b) Enzyme‐catalyzed reaction process. (c) Conversion of urea with the addition of urease in w/w and w/o systems. *Source*: Reproduced from Ref.^[70]^ with permission. Copyright (2022), American Chemical Society.

Meng et al. designed an aqueous two‐phase droplet bioreactor based on PEG and Dex phases as the continuous phase and dispersed phase for the study of enzymatic reactions (glucose oxidase, *β*‐D‐glucosidase, and urease), respectively (Figure 12a,b).^[70]^ In aqueous two‐phase system (ATPS) droplets, substrates can be distributed both inside and outside the droplet. This selective permeability allows the catalytic product to penetrate into the PEG phase, which is more similar to the activity of a cell. Aqueous substrates and products can diffuse freely with the continuous phase at the water‒water interface. In comparison, the conversion rate of a w/w droplet without the permeation effect is 63.2%, while that of a w/o droplet is 17.9% (Figures 12c). This study provides a powerful system for simulating cell material transport and is highly useful in chemical and biological research.

**FIGURE 13:**
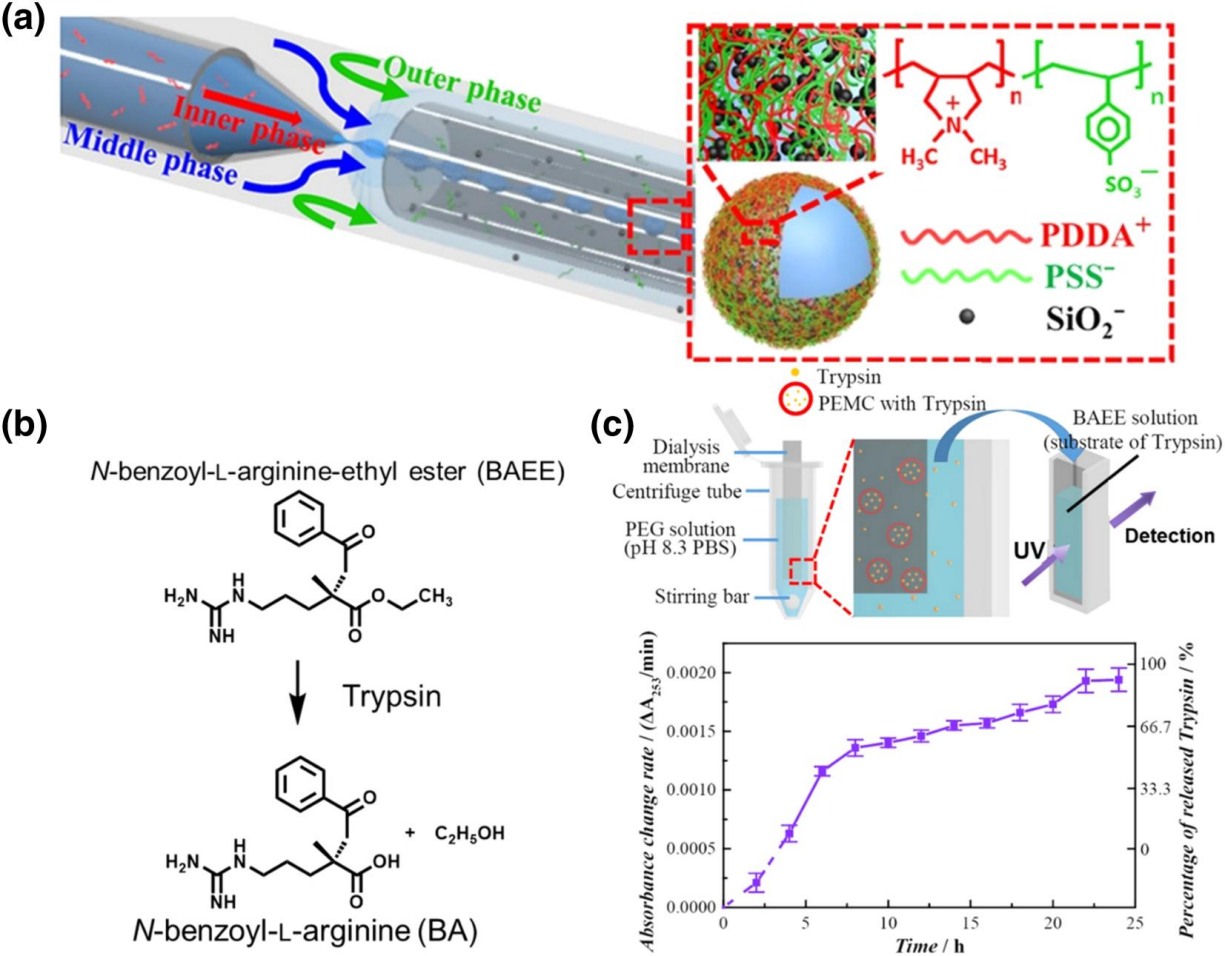
(a) Enzyme‐catalyzed reaction process. (b) Schematic of the three‐phase droplet system consisting of inner, middle, and outer aqueous phases. (c) Measured release percentage of trypsin with time. *Source*: Reproduced from Ref.^[71]^ with permission. Copyright (2019), American Chemical Society.

While microfluidic systems can be used to prepare w/w droplets for high‐throughput enzyme screening, the droplets are subject to deformation and aggregation because of their low interfacial tension and large thickness. Zou et al. designed a stable microfluidics platform consisting of a three‐phase (inner, middle, and outer) aqueous system for the formulation of stable and size‐controllable w/w emulsions (Figure 13a).^[71]^ Notably, these w/w droplets were synthesized via electrostatic interactions between positively charged poly(diallyldimethylammoniumchloride) (PDDA^+^) and negatively charged polystyrene sodium sulfate (PSS^−^), and anionic SiO_2_ nanoparticles were used as stabilizers to enhance the stability, permeability, and mechanical strength of the droplets. By controlling the osmotic pressure and pH of the droplets, the encapsulated molecules were released. Here, the trypsin‐catalyzed reaction of BAEE to form BA was studied as a model reaction (Figure 13b). The model enzyme trypsin was successfully encapsulated in the emulsion, enabling it to function for a long period of time (25 h) (Figure 13c). This study describes a method for adjusting the liquid flow rate and external mechanical perturbations to produce uniform droplets without damaging biological enzymes or denaturing biomolecules by maintaining their contact with organic solvents. In the biological and pharmaceutical fields, this study will provide a platform‐based approach for studying the encapsulation, carrying, delivery, and release of drugs.

### 5.2 Enzyme catalysis at w/o interfaces

**FIGURE 14:**
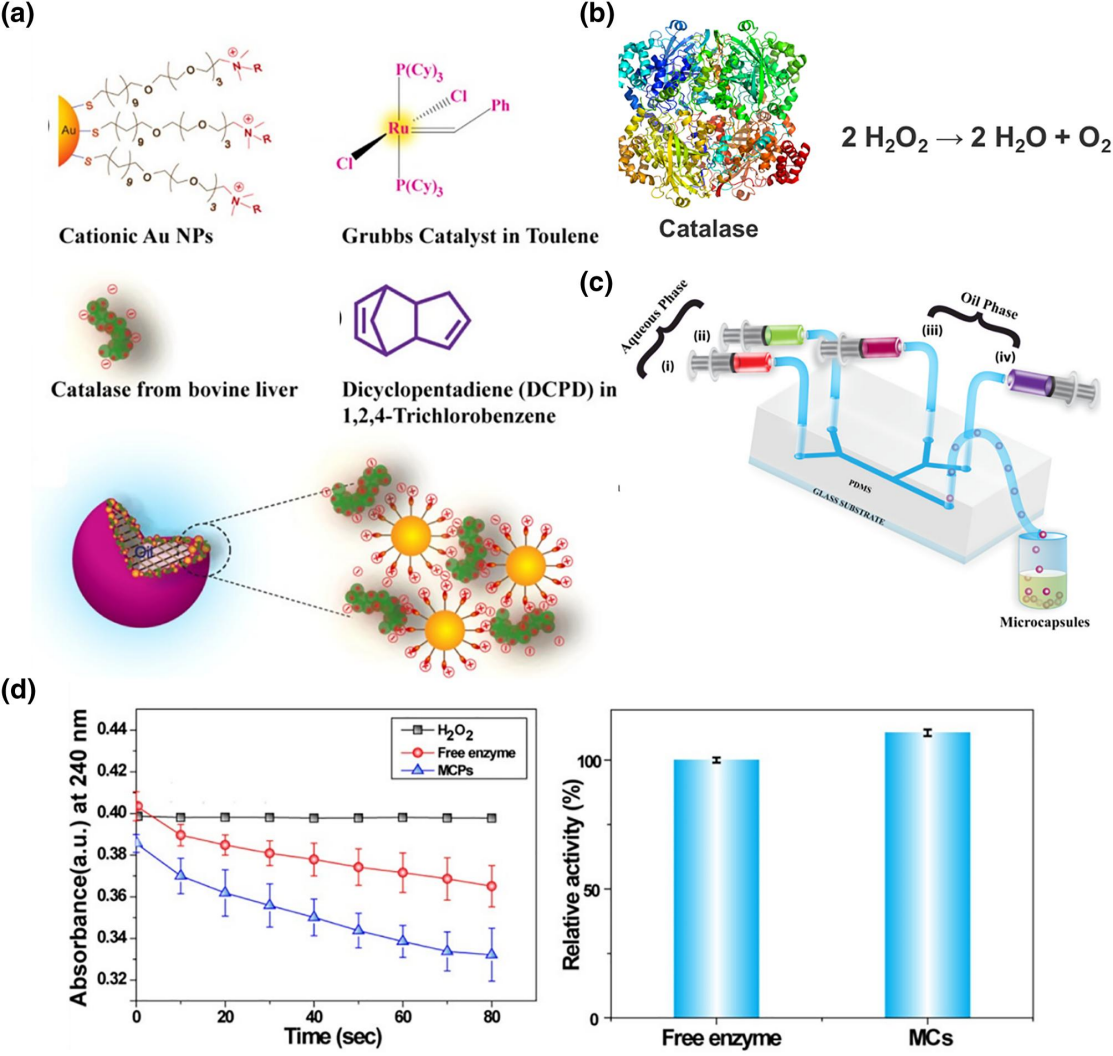
(a) Structure of the ligand used, including cationic Au NPs, Grubbs catalyst, dicyclopentadiene, and catalase from bovine liver. Schematic of the (b) enzyme catalysis process, (c) construction via electrostatic interactions, and (d) microfluidic device. *Source*: Reproduced from Ref.^[78]^ with permission. Copyright (2019), American Chemical Society.

The immobilization of enzymes is crucial to achieve improved enzyme utilization, and immiscible oil‐water interfaces are ideal for enzyme immobilization in droplets.[^72^, ^73^] In the past, extensive studies have been conducted on the development of the solid‐phase interfacial immobilization of enzymes, including binding, cross‐linking, and capturing.[^74^, ^75^, ^76^, ^77^] However, it is quite difficult to immobilize enzymes at the oil–water interfaces of droplets. To resolve this issue, Varshney et al. developed a method for immobilizing catalase at w/o interfaces by combining negatively charged enzymes with positively charged gold nanoparticles (Au NPs) and applying a Grubbs catalyst and dicyclopentadiene (DCPD) monomer as interfacial bridges (Figure 14a).^[78]^ In this work, the catalytic efficiency of free and immobilized enzymes was compared using the classic catalase model (Figure 14b). As nanoparticles and enzymes were injected into a double Y‐shaped microfluidic, noncovalent complexes formed through electrostatic interactions. Subsequently, water droplets were encapsulated by the oil phase, resulting in complexes migrating to the oil–water interface to stabilize the oil droplets (Figure 14c). In an activity comparison of immobilized and free enzymes, immobilized catalase showed 1.1 times greater activity and could be reused multiple times (Figure 14d). Thus, this study has provided an assembly method for stabilizing enzymes by nanoparticles, and the resulting monodisperse enzyme retains its activity through multiple catalytic cycles, which could be applied to other biocatalytic processes.

**FIGURE 15:**
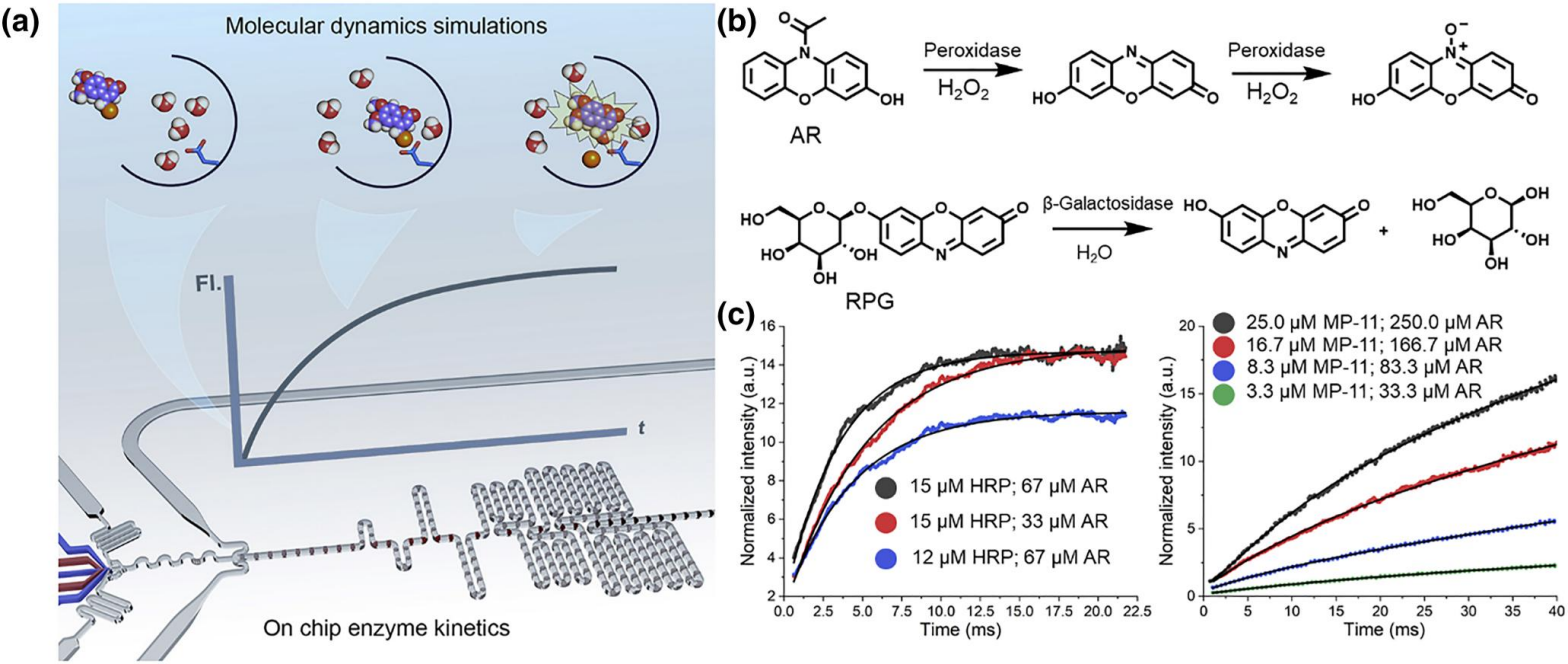
(a) Schematic of the high‐throughput acquisition of transient kinetic data with microfluidic platforms. (b) Catalytic processes of peroxidase and *β*‐galactosidase. (c) Kinetic data for the model enzymatic assays. *Source*: Reproduced from Ref.^[79]^ with permission. Copyright (2021), The Cell Press.

With their high degree of integration, compatibility, and operability, microfluidic systems can be integrated with other high‐performance instruments, providing a powerful tool for studying droplet catalysis. Stavrakis et al. recently proposed a droplet‐based microfluidics platform for the measurement of multiple enzyme reactions (peroxidase and *β*‐galactosidase) with ultrafast kinetics.^[79]^ Droplets were controlled by speed, and they enter the serpentine channel path to accelerate rapidly and cause rapid mixing (Figure 15a). In microfluidic systems, ferric microperoxidase (MP‐11)‐mediated oxidation of Ampliflu Red (AR) and *β*‐galactosidase‐mediated hydrolysis of resorufin *β*‐D‐galactopyranoside (RGP) were used to study transient kinetics and thermodynamics (Figure 15b). The kinetics of model enzyme assays indicated that resorcinol oxidation occurred rapidly, whereas the formation of resazurin was slow. Glycosidase hydrolysis is a linear reaction that does not appear in equilibrium under the conditions used (Figure 15c). This platform allowed the efficient and systematic collection of comprehensive kinetic data for the study of enzyme catalysis. This study provides new insights into the molecular basis of substrate specificity and the role of hydration‐related entropy based on high‐throughput data collection and global molecular dynamics simulations.

**FIGURE 16:**
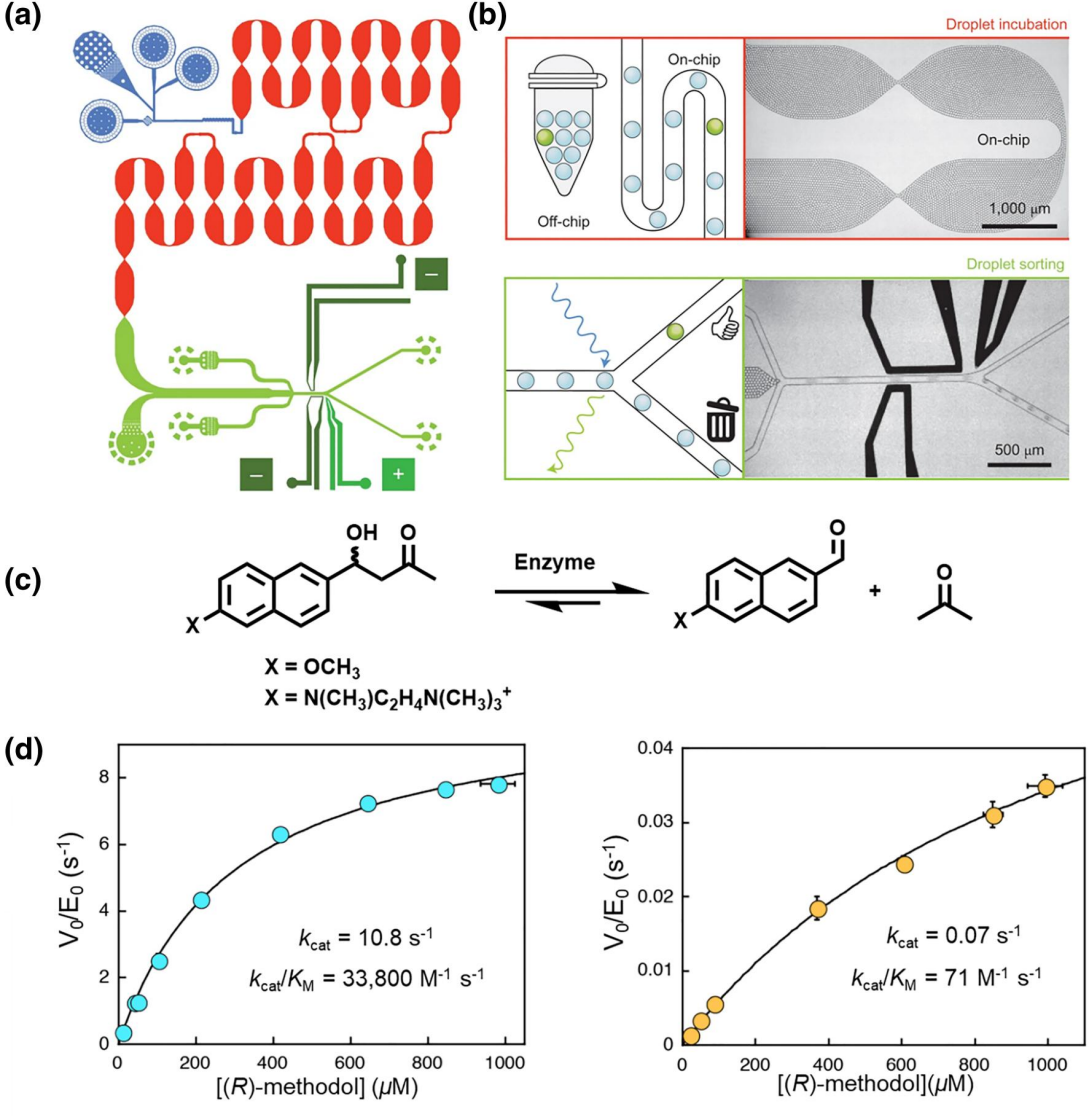
Schematics of (a) the microfluidic platform and chip designs and (b) the high‐throughput screening of shear enzyme reactions featuring droplet generation, incubation, and sorting on a single chip. (c) Enzyme‐catalyzed reaction process. (d) Michaelis‒Menten plot of enzyme for screening with racemic substrates. *Source*: Reproduced from Ref.^[80]^ with permission. Copyright (2017), Springer Nature.

Understanding the process of enzyme catalysis in living organisms is important for better simulating enzyme catalysis. By combining the flexibility of microtiter plate analysis with ultrahigh throughput fluorescence‐activated cell sorting, Obexer et al. developed independent microreactors based on droplet microfluidic systems (Figure 16a).^[80]^ Fluorescent product‐containing droplets were then sorted by a microfluidic fluorescence‐activated droplet sorter (FADS) at a rate of up to 2000 droplets per second, reducing the time required for conventional plate assays (Figure 16b). Aldolase is a large molecule that promotes the reversible cleavage of carbon‒carbon bonds to form aldehydes and enamines and has been used in biologically asymmetric syntheses. Despite screening with racemic substrates, the designed enzymes were highly selective and preferred the (*R*)‐enantiomer over the (*S*)‐enantiomer (Figure 16c,d). In addition to retaining the high activity of native aldolases, this screening method can also assist in identifying catalysts that preserve the rates and selectivity of native aldolases.

**FIGURE 17:**
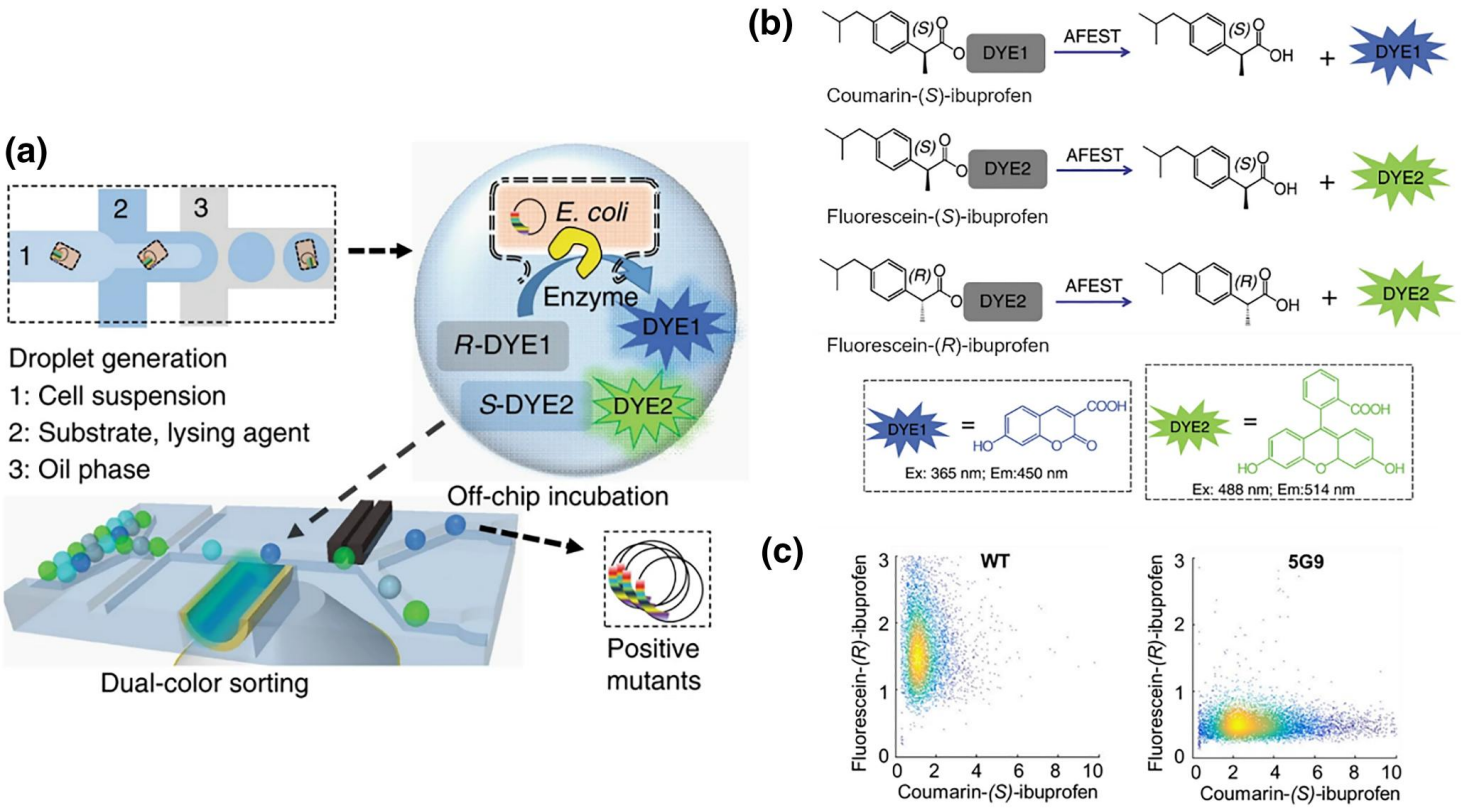
(a) Schematic of the dual‐channel microfluidic droplet screening (DMDS) platform for the directed evolution of complex enzymes. (b) Enzyme‐catalyzed reaction process. (c) Enantioselectivity of WT AFEST and 5G9 in a biased mode. *Source*: Reproduced from Ref.^[81]^ with permission. Copyright (2018), Springer Nature.

Based on a similar principle, Ma et al. reported a dual‐channel microfluidic droplet screening (DMDS) platform that enables the efficient screening of enantioselective enzymes for the directed evolution of complex enzymes.^[81]^ By using dual‐fluorescence microfluidic devices, enzymes can be detected and sorted with a high throughput in separate microreactors (Figure 17a). The anti‐inflammatory drug profens was used here as a model molecule; (*S*)‐profens acts as an anti‐inflammatory, while its enantiomer (*R*)‐profens has serious side effects. AFEST is a thermophilic esterase from *Archaeoglobus fulgidus* that cleaves aryl esters to yield enantiomers (Figure 17b). As a result of targeting AFESTs with this platform, a mutant 5G9 enzyme showed an improved activity for substrate 1 but a decreased activity for substrate 3, showing improved enantioselectivity over that of the wild‐type enzyme (Figure 17c). The screening platform enables the rapid generation of enzymes with specific properties, such as enantiospecificity, chemospecificity, and regiospecificity.

## 6 OUTLOOK OF ENZYME CATALYTIC SYSTEMS IN MICROFLUIDIC DROPLETS: CHALLENGES AND OPPORTUNITIES

A w/o droplet is an aqueous compartment surrounded by oil, which can be stabilized by surfactants or amphiphilic molecules that align themselves at the interface between the water and oil. The use of organic solvents will inevitably result in the irreversible deactivation of enzymes. However, w/w droplets can solve this problem, but their uncontrollable stability has always limited their rapid development, which makes it difficult to encapsulate multiple components within them. In addition to double emulsions, water‐in‐oil‐in‐water (w/o/w) emulsions have been developed, which require a complicated emulsification step but have great advantages in terms of stability.[^1^, ^82^, ^83^] Such w/o/w emulsions are polydisperse, and the internal aqueous compartment can be divided into multiple compartments to encapsulate a variety of substances for more complex studies, such as the in vitro evolution of *β*‐galactosidase through double emulsion fluorescence‐activated sorting^[84]^ or the determination of single‐cell lysates within emulsion droplets using double emulsion flow cytometry.^[85]^ It is therefore expected that the design of a more stable double‐emulsion system will be a future development direction and will stimulate the continued development of new enzyme‐catalyzed reactions.

As the reviewer's mentioned, this review focuses on the recent progress and representative examples of catalysis in microfluidics droplets, including homogeneous, heterogeneous, and enzyme catalysis, while other droplet‐derived systems, such as liposomes and colloidosomes, have not been mentioned in our previous manuscript due to the space limitation. However, we fully agree with the reviewer's opinion that it would be better if other droplet‐derived systems such as liposomes, colloidosomes, etc. could be discussed. Thus, the following discussion has been added in the revised manuscript.

In recent years, microfluidics has become an important tool for preparing microdroplet systems, especially in liposomes and colloidosomes.[^86^, ^87^] As one of the most widely used drug delivery platforms, liposomes are artificial sphere‐shaped vesicles composed of natural cholesterol and phospholipids. Colloidosomes are microcapsules with shells made up of colloidal particles, which can encapsulate and release active ingredients and are used in many fields, such as drug delivery and catalysis. Microfluidic chips not only can be applied for the large‐scale synthesis of stable and size‐defined monodisperse droplets due to their remarkable advantages over traditional synthesis methods,[^88^, ^89^] but also artificially controls mixing temperature, concentration, the intrinsic characteristics of liposomes, such as charge and phase transition temperatures, which make it possible to bridge the gap between the construction of artificial liposome and complex biological functions.^[90]^

By a multistep microfluidic strategy, Huck et al. prepared a series of uniform liposomes, such as concentric, pericentric, and multicompartment liposomes, which provide a paradigm for building complex artificial cell models and studying signal transmission within subcellular organelles.^[91]^ A high‐throughput microfluidic method was developed by Spatz et al. to create giant unilamellar vesicles with discrete compartments for loading different biomolecules.^[92]^ This system can be used to separate the vesicles from the oil phase to study their interaction with the real physiological environment. This method is a major advance in preparing artificial biomimetic vesicles using microfluidics. van Hest et al. prepared a Pickering emulsion stabilized by polymersomes for biphasic enzyme catalysis.^[93]^ The enzyme can be loaded into either the water phase or the polymer vesicle cavity of the Pickering emulsion, which greatly improves its catalytic performance and facilitates its recycling and reuse. Additionally, different enzymes can be added in different compartments, making this an ideal cascade reaction platform. However, the current microfluidic technology focuses on preparing liposomes and colloids. Without a doubt, the liposomes and colloids prepared by this method are an ideal platform for more complex biomimetic catalysis research.

The paradigm of biochemical reactions has been changed by microfluidic systems, especially in regard to simulating enzymatic reactions. The catalysis of biological enzymes is different from that of metal reactions because the environment is aqueous. Biological enzymes must therefore be used under strict consideration of the composition of droplets during the catalytic process so that they can exert a maximum catalytic activity in the aqueous phase or at the interface. In this paper, we reviewed the enzymatic processes of w/o and w/w in droplets. Many systems have been developed to ensure the activity of enzymes, but the following issues still need to be considered: (1) Enzymatic catalysis often involves a variety of coenzyme factors, and a simple water system cannot accurately mimic the complex cellular environment. (2) At the cell interface, enzyme catalysis also plays a significant role. Several factors contribute to catalytic reactions, including the direction of enzyme interface enrichment and arrangement. To avoid excessive density affecting enzymes and substrates, the arrangement and assembly of enzymes at the w/o interface must be considered. (3) Specificity in enzyme catalysis is key to improving the synthesis efficiency of compounds, and increasing the reuse rate of enzymes in microfluidic droplets, product separation, and cascade catalysis will lead to the development of more enzyme catalysts in the chemical synthesis.

## 7 CONCLUSION

In this review, we summarize recent developments in catalytic reactions occurring in droplets generated by microfluidics, provide a brief overview of the various reactions catalyzed by different catalytically active centers, describe the unique properties of these reactions, and highlight the application of droplets in catalytic processes (Table 1). Microfluidics provides a powerful method for controlling the size and shape of synthesized droplets. Thus, it opens a pathway for facilitating microscale chemistry.

**TABLE 1:** Characteristics of catalytic reactions that occur in microfluidic droplets.

	Microreactor	Size	Temp.	Flow rate	Catalyst	Reactants	Ref.
Homogeneous catalytic systems	PDMS	100 μm	RT	300 μm h^−1^	Tetrabutylammonium bromide	Benzyl bromide phenoxide	[32]
Homogeneous catalytic systems	PEEK	250 μm	80°C	50 μm min^−1^		Phenylacetonitrile + butyl bromide	[33]
Homogeneous catalytic systems	PTFE	500 μm	180°C	505 μm min^−1^	Iron sulfate	Methane + oxygen	[34]
Wall‐coated catalyst	Silica capillaries	500 μm	20°C	200 μm min^−1^	Nanosized Pd	Sodium + hydrogen	[40]
Packed bed catalyst	PTFE	220 μm	0°C	100 μm min^−1^	AO‐TEMPO packed beds	Primary and secondary alcohols	[42]
Colloidal nanoparticle	PTFE	4.76 mm	RT	276 μm min^−1^	Nanosized Pt	Nitrobenzene	[44]
Pes in microreactors	PTFE	15 μm	60°C	25 μm min^−1^	HCl	Benzaldehyde dimethyl acetal	[51]
Slurry catalysis	PDMS	500 μm	90°C	6 μm h^−1^	Zeolites	Anisole	[52]
Enzyme catalysis	PTFE	200 μm	20°C	10 μm min^−1^	Immobilized xylanase	Xylan	[69]
Enzyme catalysis	Glass capollaries	255 μm	RT	1 μm min^−1^	Urease	Urea	[70]
Enzyme catalysis	PDMS	400 μm	RT	250 μm min^−1^	Gold nanoparticle—catalase	Hydrogen peroxide	[78]
Enzyme catalysis	PDMS	100 μm	20°C	22 μm min^−1^	Haloalkane dehalogenase	1, 2‐dibromoethane	[80]
Enzyme catalysis	PDMS	45 μm	RT	50 μm min^−1^	Thermophilic esterase	(*S*)‐profens	[81]

Abbreviations: AO‐TEMPO, AMBERZYME® oxirane‐tetramethylpiperidine‐1‐oxyl; PDMS, polydimethylsiloxane; PEEK, poly(ether ether ketone); PTFE, poly tetra fluoroethylene.

Although numerous studies have focused on catalytic reactions, far fewer studies have focused on the confined space of droplets, leaving a large research area to be explored. It is still challenging to fabricate these droplets and promote facile processes. The size of the droplets fabricated using microfluidic techniques is usually large, which limits their efficiency when used for catalysis. Future research needs to focus on the production of very small droplets down to the nanoscale. In addition to oil–water systems, host‐guest molecular recognition can also be extended to ATPSs, which shows promising applications in biology, cosmetics, and food. However, the catalytic functions and types of catalytic reactions are still limited. Therefore, the use of computational methods can help design substrates artificially, rather than constantly searching for new substrates, which will make reactions more targeted. Addressing these issues will benefit the production of a new generation of catalytic systems with novel functionalities.

## CONFLICT OF INTEREST STATEMENT

The authors declare no conflict of interests.
